# Direct measurement of discrete valley and orbital quantum numbers in bilayer graphene

**DOI:** 10.1038/s41467-017-00824-w

**Published:** 2017-10-16

**Authors:** B. M. Hunt, J. I. A. Li, A. A. Zibrov, L. Wang, T. Taniguchi, K. Watanabe, J. Hone, C. R. Dean, M. Zaletel, R. C. Ashoori, A. F. Young

**Affiliations:** 1 Department of Physics, Massachusetts Institute of Technology, Cambridge, MA 02139 USA; 2 Department of Physics, Columbia University, New York, NY 10027 USA; 3 Department of Physics, Carnegie Mellon University, Pittsburgh, PA 15213 USA; 4 Department of Physics, University of California, Santa Barbara, CA 93106 USA; 5 Department of Mechanical Engineering, Columbia University, New York, NY 10027 USA; 6 Advanced Materials Laboratory, National Institute for Materials Science, Tsukuba, Ibaraki 305-0044 Japan; 7 Station Q, Microsoft Research, Santa Barbara, CA 93106-6105 USA

## Abstract

The high magnetic field electronic structure of bilayer graphene is enhanced by the spin, valley isospin, and an accidental orbital degeneracy, leading to a complex phase diagram of broken symmetry states. Here, we present a technique for measuring the layer-resolved charge density, from which we directly determine the valley and orbital polarization within the zero energy Landau level. Layer polarization evolves in discrete steps across 32 electric field-tuned phase transitions between states of different valley, spin, and orbital order, including previously unobserved orbitally polarized states stabilized by skew interlayer hopping. We fit our data to a model that captures both single-particle and interaction-induced anisotropies, providing a complete picture of this correlated electron system. The resulting roadmap to symmetry breaking paves the way for deterministic engineering of fractional quantum Hall states, while our layer-resolved technique is readily extendable to other two-dimensional materials where layer polarization maps to the valley or spin quantum numbers.

## Abstract (web summary)

The phase diagram of bilayer graphene at high magnetic fields has been an outstanding question, with orders possibly between multiple internal quantum degrees of freedom. Here, Hunt et al. report the measurement of the valley and orbital order, allowing them to directly reconstruct the phase diagram.

## Introduction

The single-particle energy spectrum of a two-dimensional electron system (2DES) in a large magnetic field collapses into Landau levels (LLs) containing *N* ^Φ^ degenerate states, with *N* ^Φ^ the number of magnetic flux quanta penetrating the sample. The width in energy of the LL bands is limited only by disorder, making electronic interactions effectively strong in a clean system even when their absolute scale is weak. The simplicity of the starting LL wavefunctions, combined with the high degree of control available in 2DES, make LLs a promising venue for engineering electronic ground states based on electron correlations. However, the difficulty of simulating interacting electron problems necessitates experimental input to constrain the possible ground states, particularly in the presence of internal degeneracy. The Bernal bilayer graphene (B-BLG) zero energy Landau level (ZLL) provides an extreme example of such degeneracy. In B-BLG, LLs $\left| {\xi N\sigma } \right\rangle$ are labeled by their electron spin *σ* = ↑, ↓, valley *ξ* = +, −, and orbital index $N \in {\Bbb Z}$. Electrons in valley +/− are localized near points *K*/*K*′ of the hexagonal Brillouin zone, while the index *N* is closely analogous to the LL-index of conventional LL systems. The energies of the LLs are approximately ${\epsilon _{\sigma \xi N}} \approx \hbar {\omega _c}{\rm{sign}}\left( N \right)\sqrt {N\left( {N - 1} \right)}$, where *ħω*_c_ is the cyclotron energy, leading to an eight-fold nearly degenerate ZLL comprising the *N* = 0 and *N* = 1 orbitals and all possible spin and valley isospin combinations.

While direct probes of spin in 2DESs were demonstrated two decades ago^1^, the valley quantum number has only been probed indirectly in semiconductor quantum wells^2^, graphene monolayer^3, 4^ and bilayers^5–13^, and transition metal dichalcogenides^14^. In bilayer graphene, resolving the order in which the eight components fill as electrons are added is one of the key open questions, and is essential to efforts to use bilayer graphene to engineer exotic phases of matter based on electronic correlations^15, 16^. Due to an approximate SU(4) symmetry relating spin and valley, determining which of the components are filled is non-trivial. Past experiments^5–13, 17^ have observed numerous phase transitions between gapped ground states at both integer^6–9, 12, 13, 17^ and fractional^10, 11, 13^ filling. However, these experiments are insufficient to constrain realistic theoretical models, in which the preferred ordering is determined by a combination of the Zeeman energy, which splits the spins; Coulomb interactions and band structure effects, both of which distinguish between the *N* = 0, 1 orbitals; and several small valley anisotropies which weakly break the valley-SU(2) symmetry^18–24^. Indeed, two recent experimental papers explain their data using mutually contradictory single-particle^12^ and purely interacting pictures^10^. Constructing a more complete theory of symmetry breaking in bilayer graphene requires experimental determination of the partial filling of each spin, valley, and orbital level, ${\nu _{\xi N\sigma }} = \langle {\hat N_{\xi N\sigma }^e} \rangle {\rm{/}}{N^\Phi }$ as it evolves with total LL filling.

In this work, we introduce a direct measurement of two out of three of these components, by exploiting the fact that the four valley and orbital components indexed by *ξN* have different weights on the two layers of the bilayer. We detect this difference in layer polarization capacitively, and use it to infer the fillings *ν*_*ξN*_ as a function of both the total electron density and applied perpendicular electric field.

## Results

### Layer polarization measurements

**Fig. 1:**
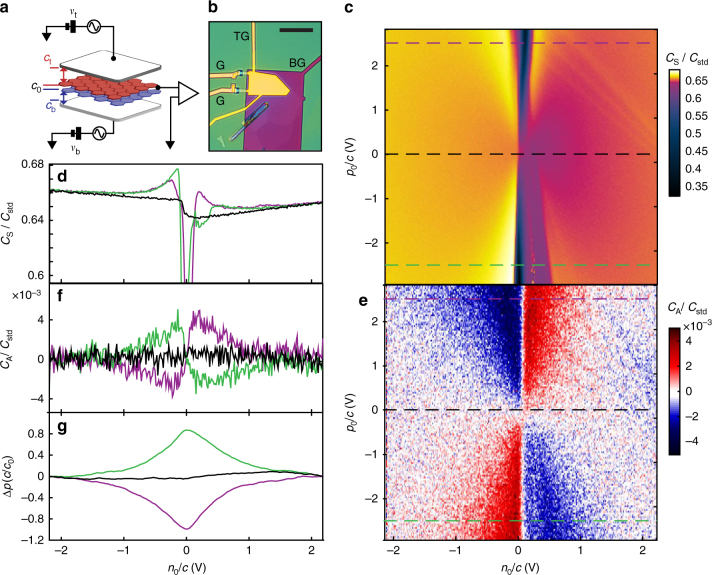
Layer polarization of bilayer graphene at zero magnetic field. **a** Measurement schematic showing geometric gate capacitances *c*_t_ and *c*_b_ and interlayer capacitance *c*_0_. Capacitance is measured using a cryogenic bridge circuit by comparison with a standard capacitor *C*_std_, measured to be 404 ± 20 fF (see “Methods”). **b** Device image. Top gate (TG), back gate (BG), and contacts to bilayer graphene (G) are shown. *Scale bar* is 10 μm; device area is approximately 87 μm^2^. **c** *C*_S_ measured at *B* = 0 and *T* = 1.6 K as a function of *n*_0_/*c* = *v*_t_ + *v*_b_ and *p*_0_/*c* = *v*_t_ − *v*_b_. A *p*_0_-dependent band gap is visible as the dark region near *n*_0_ = 0. **d** Line traces taken at different values of *p*_0_, corresponding to *dashed lines* in **c**. Band edge van Hove singularities^28^ and electron-hole asymmetry^27^ are both evident. **e** *C*_A_ measured under the same conditions. A common, constant background has been subtracted to account for fixed parasitic capacitances. **f** Line traces at different values of *p*_0_ corresponding to *dashed lines* in **e**. **g** Integrated change in polarization, $\frac{{{c_0}}}{c}{\int} {{C_A}{\kern 1pt} d\left( {\frac{{{n_0}}}{c}} \right) = \Delta p}$, with the constant of integration fixed to be zero at high $\left| {{n_0}} \right|$. In accordance with single-particle band structure^28^, wavefunctions are layer unpolarized for *p*_0_ = 0, while for large $\left| {{p_0}} \right|$ the polarization peaks at *n*_0_ = 0, where band wavefunctions are strongly layer polarized

Our devices consist of hexagonal boron nitride encapsulated B-BLG flakes^25^ fitted with metal top and bottom gates (Fig. 1a, b). The layer polarization and total charge density are tuned by a combination of applied top and bottom gate voltages (*v*_t_ and *v*_b_), expressed through their symmetric and antisymmetric combinations *n*_0_(*p*_0_) ≡ *c*_t_ *v*_t_ ± *c*_b_ *v*_b_ with *c*_t(b)_ the geometric capacitances between the respective gates and the B-BLG. −*n*_0_ and −*p*_0_ correspond to the induced charge density and layer polarization in the limit of a perfectly metallic, infinitesimally spaced bilayer. Generically, the physically realized total density (*n*) and layer density imbalance (*p*) deviate from this limit, particularly at high magnetic fields. A simple electrostatic model (Supplementary Note 1) shows that these deviations manifest as corrections to the measured gate capacitances (*C*_T_ and *C*_B_) as

1 $${C_{\rm{S}}} \equiv {C_{\rm{T}}} + {C_{\rm{B}}} = 2c\frac{{\partial n}}{{\partial {n_0}}}$$

2 $${C_{\rm{A}}} \equiv {C_{\rm{T}}} - {C_{\rm{B}}} = 2c\frac{{\partial n}}{{\partial {p_0}}} = \frac{{{c^2}}}{{{c_0}}}\frac{{\partial p}}{{\partial {n_0}}},$$

where *c* = (*c*_t_ + *c*_b_)/2 ≈ 1.36 fF/μm^2^ and *c*_0_ is the interlayer capacitance of the bilayer. The antisymmetric combination *C*_A_ is unique to bilayer systems, vanishing identically in a monolayer system, allowing us to measure the layer polarization *p*. Measuring *C*_A_ is technically challenging, as it arises from series combination of the large interlayer capacitance and the comparatively small gate capacitance. It is imperative that the dielectric layers be highly uniform across the device, that the areal mismatch between top and bottom gate be kept $\ll 1\%$, and the geometric capacitance of the two gates be nearly the same; these requirements are difficult to satisfy in conventional two-dimensional electron bilayers but are readily achieved using single-crystal hBN gate dielectrics and atomically thin bilayers.

Figure 1c and e show *C*_S_ and *C*_A_ measured at zero magnetic field as a function of *n*_0_ and *p*_0_. The *C*_S_ data are dominated by quantum capacitance features of the B-BLG band structure, which features a quadratic band touching at low energies and hyperbolic bands at high energies^26^. An electric field (*p*_0_) opens a band gap with $\sqrt \epsilon$ van Hove singularities at the band edges, as can be readily seen in the experimental data (Fig. 1d). Although *C*_S_ is approximately particle-hole symmetric, significant symmetry breaking contributions are evident. We attribute this to the skew interlayer hopping parameter *γ*_4_ in the Slonczewski-Weiss-McClure model for graphite, which breaks particle-hole symmetry by making the lattice non-bipartite (Supplementary Fig. 4)^27^. *C*_A_ data, in contrast, reflect the layer-resolved properties of the band wavefunctions (Fig. 1e, f). For *p*_0_ ≠ 0, wavefunctions are layer-polarized near the band extrema, so that the first electrons or holes added to the neutral system are added to the corresponding low-energy layer. Reversing *p*_0_ inverts the role of the top and bottom layers, inverting the sign of the measured signal with respect to *n*_0_. At high overall electron density, the applied *p*_0_ is fully screened, so that charge is added symmetrically to the two layers^28^. The relative layer polarization at different values of *n*_0_ can then be extracted by integrating Eq. (2) with respect to *n*_0_ (Fig. 1g).

### Layer polarization at high magnetic field

**Fig. 2:**
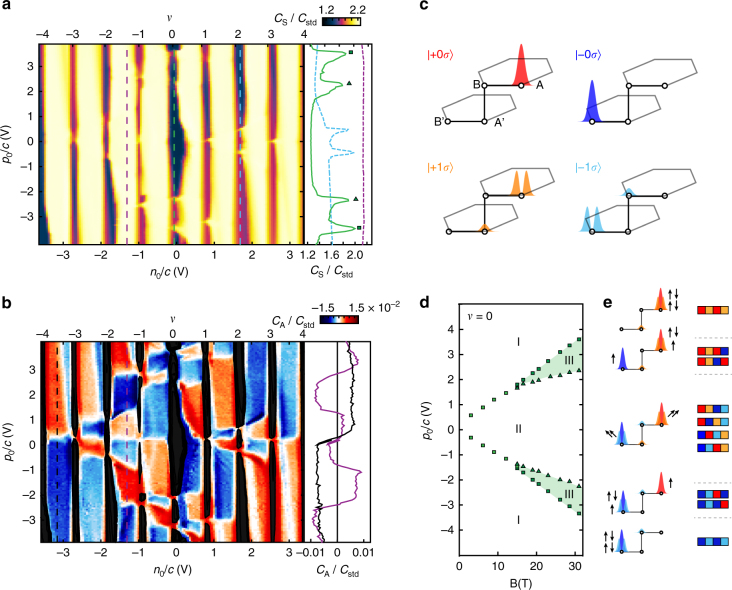
Valley and orbital polarization of the ZLL. **a** Layer-symmetric capacitance ${C_{\rm{S}}} \propto \frac{{\partial n}}{{\partial {n_0}}}$ at *T* = 300 mK and *B* = 31 T. Incompressible states manifest as drops in *C*_S_ (*black*) at all integer fillings *ν*. Phase transitions between different valley and orbital fillings at fixed *ν* manifest as compressible spikes, as shown in the side panel for *ν* = 0 (*green*, *solid*) and *ν* = 2 (*light blue*, *dashed*). A total of 16 phase transitions are observed at integer *ν*, with one each at *ν* = ±3, two at *ν* = ±2, three at *ν* = ±1, and four at *ν* = 0. No experimental contrast is visible at non-integer filling (*purple*, *dashed*). **b** Layer-antisymmetric capacitance ${C_{\rm{A}}} \propto \frac{{\partial p}}{{\partial {n_0}}}$ at *T* = 300 mK and *B* = 31 T. *Black* regions mask portions of the parameter space with large dissipation in *C*_S_, which arises when a large gap leads to a low in-plane conductivity and failure to charge regions of the sample during a ~13 μs measurement cycle (see “Methods”). The color scheme highlights the 4-tone contrast, interpreted as filling of $\left| {\xi N\sigma } \right\rangle$ = $\left| { + 0\sigma } \right\rangle$ (*red*), $\left| { + 1\sigma } \right\rangle$ (*orange*), $\left| { - 0\sigma } \right\rangle$ (*blue*), and $\left| { - 1\sigma } \right\rangle$ (*cyan*) LLs. **c** Schematic depiction of the four single-particle wavefunctions |*ξNσ*〉, showing their relative support on the four atomic sites *A*, *B*, *A*′, and *B*′ of the bilayer graphene unit cell. While the $\left| { + 0\sigma } \right\rangle$ levels are fully polarized (*α*_0_ = 1), we calculate *α*_1_ = 0.63 for the $\left| { + 1\sigma } \right\rangle$. **d** Phase diagram of gapped states at *ν* = 0. Points are experimentally determined by measuring peaks in *C*_S_, as in **a** (*green dashed line*), for 0 < *B* < 31 T. At high *B* ≳ 15 T an intermediate phase III emerges between the layer-unpolarized canted antiferromagnetic phase II and the layer-polarized phase I^6, 8^. **e** Schematics of the layer, orbital, and spin polarizations of phases I, II, and III and the ten distinct filling sequences that determine the three valley and orbital polarizations of phases I, II, and III. These sequences are extracted from Fig. 2b, filling from *ν* = −4 to *ν* = 0 over the full range −4*V* < *p*_0_/*c* < 4*V*

Figure 2a shows *C*_S_ measured in the same device at *B* = 31 T in the ZLL. We observe insulating states at all integer LL filling factors *ν*, which are characterized by low capacitance *C*_S_ and high dissipation (see Supplementary Figs. 1, 2 for dissipation data). Adjusting *p*_0_/*c* at fixed integer *ν* drives transitions characterized by a spike in *C*_S_ indicating increased conductivity^11^ and compressibility, consistent with a closing of the charge gap. Sixteen such phase transitions are evident in the *C*_S_ data. Similar transitions have been reported in the literature: ref. ^6^ reported phase transitions at *ν* = ±2 and *p*_0_ = 0, as well as a single phase transition at *ν* = 0 and finite *p*_0_. More recently, the *p*_0_ = 0 transitions at *ν* = ±1, ±3 are evident in ref. ^11^, while the splitting of the *p*_0_ = 0 phase transition at *ν* = ±2 suggests the formation of a stable, gapped, layer unpolarized state in the region *p*_0_ ~0, as was reported in refs. ^9, 11^. Only in ref. ^12^ was a potential gapped phase observed at intermediate *p*_0_ and *ν* = 0. However, a unified framework for understanding the diverse competing phases has not yet emerged.

In contrast to layer-insensitive capacitance^5, 10, 12^ and transport^6–9, 11^ measurements, *C*_A_ provides high experimental contrast throughout the *n*_0_ − *p*_0_ plane (Fig. 2b). Strikingly, the measured *C*_A_ falls into discrete levels, corresponding to *blue*, *cyan*, *orange*, and *red* on the color scale of Fig. 2b. The transitions observed in *C*_S_ at integer filling fall on lines in the *n*_0_−*p*_0_ plane along which the sign of *C*_A_ changes abruptly. To understand the color scale quantitatively, we compute the layer polarization of the ZLL single-particle eigenstates. B-BLG has a 4-site unit cell, with sites *A*,*B* in the top layer and *A*′, *B*′ in the bottom (see Fig. 2c), and hence the ZLL wavefunctions decompose into their components on the four sublattices, **Ψ**(*x*) = (*ϕ*_*A*_(*x*), *ϕ*_*B*′_(*x*), *ϕ*_*B*_(*x*), *ϕ*_*A*′_(*x*)). The layer polarization ${\alpha _{\xi N\sigma }} \equiv {\int} {{d^2}x\left| {{\phi _A}{{(x)}^2}} \right| + \left| {{\phi _B}{{(x)}^2}} \right| - \left| {{\phi _{A'}}{{(x)}^2}} \right| - \left| {{\phi _{B'}}{{(x)}^2}} \right|}$ is constant across all states in a LL, and independent of spin. It has opposite sign in the two valleys^26^, so that positive and negative *C*_A_ correspond to filling valley *K* and *K*′, and its magnitude depends on the orbital quantum number, so that *α*_*ξNσ*_ = *ξα*_*N*_. At *B* = 31 T, band structure calculations^29^ show that *α*_0_ = 1 and *α*_1_ = 0.63.

As electrons enter LL $\left| {\xi N\sigma } \right\rangle$ they contribute a polarization whose magnitude and sign depend on the level being filled. Since ${C_A} \propto \frac{{\partial p}}{{\partial {n_0}}}$, where *n*_0_ is very nearly the electron density, we thus interpret red, orange, blue, and cyan as indicating regions where electrons are filling $\left| {\xi N} \right\rangle$ = $\left| { + 0} \right\rangle$, $\left| { + 1} \right\rangle$, $\left| { - 0} \right\rangle$, and $\left| { - 1} \right\rangle$ type LLs, respectively. This supports a scenario in which, away from phase boundaries, only one of these LLs is filling at each particular (*n*_0_, *p*_0_). Indeed, numerical calculations (see Supplementary Fig. 5) show that as isospin *ξσ* fills, around 90% of the electrons enter into either the *N* = 0 or *N* = 1 orbital; e.g., either $\frac{{\partial {\nu _{\xi 0\sigma }}}}{{\partial \nu }}{\kern 1pt}$ ≳ 0.9 or $\frac{{\partial {\nu _{\xi 1\sigma }}}}{{\partial \nu }}{\kern 1pt}$ ≳ 0.9, according to whether the region is red/blue or orange/cyan, respectively.

The polarization of all gapped integer states can now be obtained by summing the level filling sequence starting from the *ν* = −4 vacuum. Consider *ν* = 0, where five incompressible states are visible in *C*_S_ (Fig. 2d). The order in phase I, at large *p*_0_ > 0, can be inferred from the observed *C*_A_ sequence of red, orange, red, orange, implying two *N* = 0 and two *N* = 1 states are filled in valley *ξ* = +. Its layer-inverse occurs in valley *ξ* = − for large *p*_0_ < 0. The phase I states are fully valley polarized, and hence spin and orbitally unpolarized due to Pauli exclusion. Phase II, at *p*_0_ near zero, fills levels $\left| { + 0} \right\rangle ,\left| { + 1} \right\rangle ,\left| { - 0} \right\rangle ,\left| { - 1} \right\rangle$. A state which fills both orbitals of opposite valleys is consistent with the canted anti-ferromagnetic state^8, 22^; however, from *C*_A_ alone we cannot infer whether the spins are polarized or canted. Finally, phase III at intermediate $\left| {{p_0}} \right|$ fills levels ±0, ±1, ±0, ∓0.

## Discussion

**Fig. 3:**
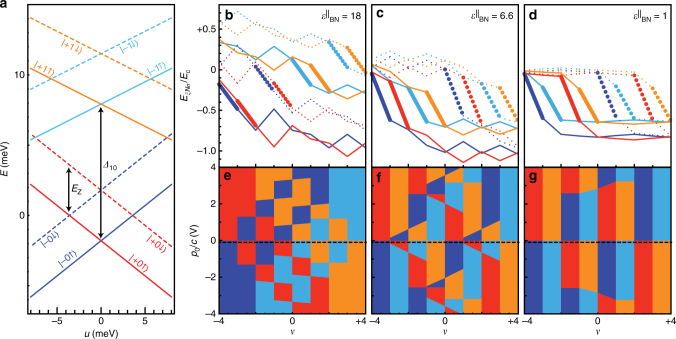
Theoretical model of spin, valley, and orbital anisotropies in the ZLL. **a** Single-particle energy spectrum of the ZLL at *B* = 31 T derived from a four-band tight binding model^29^ (see also Eq. (3)). The interlayer potential *u* couples to the layer polarization of each state as *ξα*_*N*_ *u*, differing in sign for the two valleys and magnitude for the two orbitals; the spin degeneracy is lifted by the Zeeman energy *E*_Z_ ≈ 3.6 meV; and the *N* = 0 and 1 orbitals are split by the band structure parameter Δ_10_ ≈ 9.7 meV. **b**–**d** Level filling schematic for $\frac{{{p_0}}}{c} = - 100$ mV. Within the Hartree-Fock approximation, we calculate the energy to add an additional electron to level *σξN* given the current filling {*ν*_*σξN*_}, generating eight curves *ε*_*σξN*_(*ν*) which change with the total filling *ν*. *Colors* indicate the *ξN* index of the level, while *solid* vs. *dashed* indicates the spin. The *bold portion* indicates the range of *ν* over which the level is coincident with the Fermi energy. As isospin *σξ* fills, both of its *N* = 0 and 1 orbitals decrease in energy due to favorable Coulomb correlations, while the components of the opposite valley (i.e., layer) decrease slightly in energy due to the capacitance of the bilayer. The relative magnitude of these effects, combined with the single-particle splittings, determines the filling order, shown here for three interactions strengths parameterized by the boron nitride dielectric constant, $\epsilon _{{\rm{BN}}}^{||}$. Large $\epsilon _{{\rm{BN}}}^{{\rm{||}}} = 18$ (**e**) corresponds to negligible Coulomb interactions, $\epsilon _{{\rm{BN}}}^{{\rm{||}}} = 6.6$ (**f**) corresponds to intermediate Coulomb interactions, and $\epsilon _{{\rm{BN}}}^{||} = 1$ (**g**) corresponds to maximally strong Coulomb interactoins. **e**–**g** Hartree-Fock phase diagram in the three interaction strength regimes. Colors *blue*, *cyan*, *red*, and *orange* indicate whether levels of type *ξN* = −0, −1, +0, +1 are filling, so that the result should mimic the observed *C*_A_. The intermediate interaction regime shows good agreement with the experimental *C*_A_ data, while interactions which are too weak (**e**) or strong (**g**) do not reproduce the observed filling sequences. The *black dashed line* indicates a cut at *p*_0_/*c* = −100 meV corresponding to the particular filling sequence shown in **b**–**d**

The orbital and valley filling sequences derived from Fig. 2b provide a more stringent set of constraints on theoretical models than the integer phase transitions alone. For example, a single particle, four-band tight-binding model accounts for the correct number of integer *ν* transitions^12^ but fails when compared to the *C*_A_ filling sequence. The single-particle energies of the ZLL,

3 $$E_{\xi N\sigma }^{(1)} = - {E_Z}\sigma + N{\Delta _{10}} - \frac{u}{2}\xi {\alpha _N}$$

are shown in Fig. 3a for *B* = 31 T. Here, *E*_Z_ is the Zeeman energy, *u* is the potential across the bilayer, and Δ_10_ ∝ *γ*_4_ *B* is the splitting between the *N* = 0, 1 orbitals, which arises because particle-hole symmetry no longer pins the ZLL energies to zero. The interlayer bias *u* ∝ *p*_0_ couples to the differing layer polarization of each state, *ξα*_*N*_, leading to *u*-tuned crossings at integer fillings; however, the single-particle picture predicts that *N* = 0 levels fill first for all but the very highest *u* (Fig. 3b, e), and the transitions would have zero slope in the *n*_0_ − *p*_0_ plane. This disagrees with the experimental *C*_A_ data where *N* = 0 and *N* = 1 states of the same valley often fill in sequence, and several transition lines have a significant slope.

The departure from the single-particle picture arises from the failure to account for the Coulomb interaction, which both favors sequential filling of *N* = 0, 1 levels of the same isospin due to exchange^18, 19^ and penalizes valley polarization due to the interlayer capacitance of the bilayer. However, if Coulomb correlations are made too strong, the first effect dominates and *N* = 0, 1 orbitals of an isospin always fill sequentially, as shown in Fig. 3d and g. Apparently, competition between the splitting Δ_10_ and Coulomb correlations is essential.

To determine whether a single model can account for all the observed phase boundaries, we analyze a model that accounts for the single-particle splittings, the SU(4)-invariant screened Coulomb interaction, and several subleading valley anisotropies (Supplementary Note 2). Evaluating the model within a Hartree-Fock approximation allows us to compute the energies of the competing filling sequences, and thereby determine their phase boundaries in the *n*_0_ − *p*_0_ plane. The model depends on three phenomenological constants (a screening strength, the perpendicular dielectric constant of the BLG, and a valley anisotropy), which we can now estimate by matching the location of the integer transitions and their dependence on an in-plane *B*-field. The resulting phase diagram is shown in Fig. 3c, and shows good agreement with the *C*_A_ data of Fig. 2b, including the location of the transitions in absolute units of *p*_0_/*c* and several of their slopes. In our model, each integer state is obtained by fulfilling some number of *ξNσ* levels; in particular it does not require interlayer coherent phases that spontaneously break the valley U(1) × U(1) symmetry, in contrast to theories that predict such phases at *ν* = ±1, *p*_0_ = 0. We also predict that the *ν* = 0 phase III observed at *ν* = 0 likely hosts helical edge states similar to those recently described in twisted bilayer graphene^30^. This state is stabilized by the single-particle anisotropy Δ_10_ and antagonized by the Coulomb interactions, suggesting it could be further stabilized in devices with stronger screening due to proximal metal gates.

**Fig. 4:**
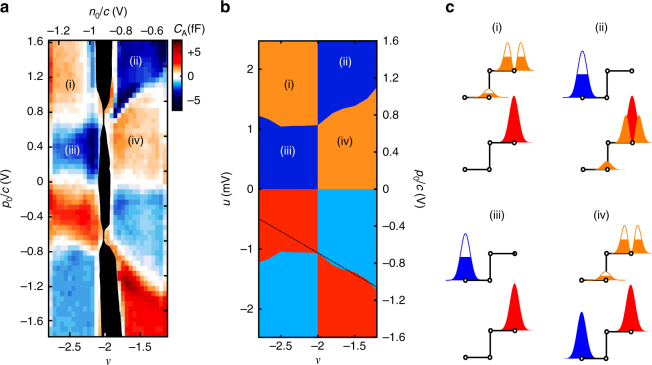
Correlation effects at partial LL filling. **a** Detail of *C*_A_ near *ν* = −2. Sign changes in *C*_A_ as a function of *n*_0_ indicate polarization extrema and phase transitions. The strong dependence of the phase transition lines on *ν* are a consequence of interactions, which cause the energy per particle of the competing phases to depend on *ν*. **b** Phase diagram from multicomponent infinite DMRG calculations^31^ for a four-band model of bilayer graphene with Coulomb interactions. In contrast to the Hartree-Fock prediction shown in Fig. 3c, experiments show that the magnitude of the slope of the phase boundary between (i) and (iii) differs from the boundary betweem (ii) and (iv). This implies that strong scattering between the *N* = 0, 1 orbitals breaks the particle-hole symmetry *ν* ↔ −(*ν* + 2), an effect which is correctly accounted for in our DMRG simulations. **c** Schematic representation of the four phases appearing in **a**. Each of the four orbital types *ξN* depicted in Fig. 2c can either be filled (*solid*, shown in *bottom unit cell*), or in the process of filling (*partially solid*, shown in *top unit cell*). Phase (ii), for example, consists of fully filled $\left| { + 0} \right\rangle$ and $\left| { + 1} \right\rangle$ levels, while level $\left| { - 0} \right\rangle$ is filling

Despite good overall agreement, there is an interesting qualitative discrepancy between the Hartree-Fock analysis and the data. In the experiment, the slope of the phase transition line between −2 < *ν* < −1 (see Fig. 4a, boundary (ii)–(iv)) is significantly larger than the slope of the adjoining transition across −3 < *ν* < −2 (boundary (i)–(iii) of Fig. 4a). Within the Hartree-Fock approximation, the slopes are identical, and in fact any model which neglects scattering (e.g., “Landau-level mixing”) between the *N* = 0, 1 orbitals has a particle-hole symmetry *ν* + 2 → −(*ν* + 2), *u* − u_*_(*ν* = −2) → −(*u* − u_*_(−2)), forcing the two boundaries to mirror each other. To account for the asymmetry, we instead find the ground state of the model’s Hamiltonian using the multicomponent infinite-density matrix renormalization group, which takes full account of correlations^31^. Allowing LL mixing produces a kink in the slope at *ν* = −2, as experimentally observed (Fig. 4b). LL mixing is known to generate effective three-body interactions, which stabilize fractional non-Abelian phases^32, 33^; our results suggest that these interactions may be stronger in BLG than in conventional semiconductor quantum wells.

In conclusion, we have described a new experimental technique to determine the layer polarization of van der Waals bilayers and used it to constrain a detailed model of symmetry breaking in the bilayer graphene ZLL. Our technique is readily applicable to quantitatively probe layer, valley, and spin polarization in other atomic layered materials, including twisted bilayer graphene and both homobilayer and heterobilayer of transition metal dichalcogenides.

## Methods

### Sample preparation

Bilayer graphene samples encapsulated in hexagonal boron nitride were fabricated using a dry transfer method^25^. Particular care is taken to ensure that the top and bottom hBN flakes are of the same thickness, measured by atomic force microscopy to be 19 and 20 nm, respectively. During fabrication, care is also taken to minimize the area of graphene bilayer gated by only one of the two gates, as single-gated areas contribute a systematic error to the measured *C*_A_ signal proportional to the area and to *C*_S_. Anticipating $\frac{{{C_A}}}{{{C_S}}} \approx \frac{c}{{2{c_0}}}$ ≲ $\frac{{3.35\,{\rm{A}}}}{{39\,{\rm{nm}}}} = .0086$, we ensure that the areal mismatch between bottom-gated and top-gated areas is less than .5%.

### Capacitance measurement electrical schematic

**Fig. 5:**
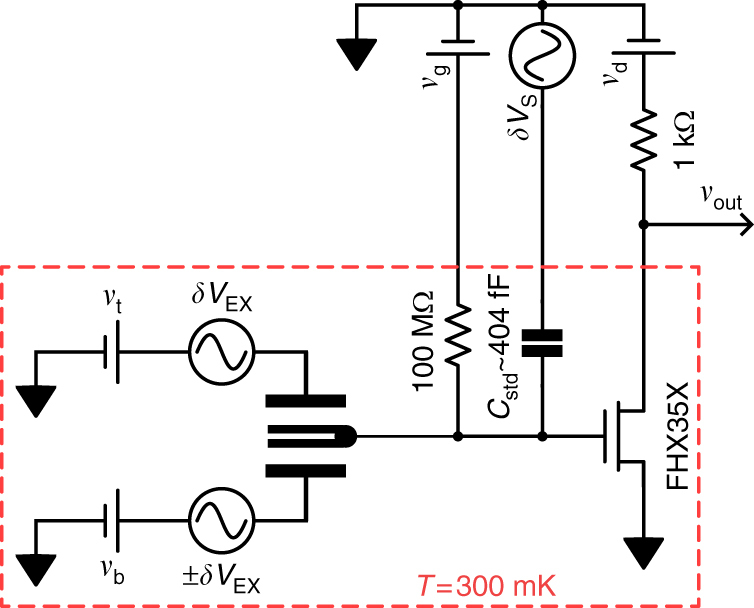
Electrical schematic of the capacitance measurement. DC voltages *v*_t_, *v*_b_ and *v*_g_ together control *n*_0_ and *p*_0_, with fixed *v*_g_ ≈ −300 mV such that the transistor amplifier is at its optimal working point. For *C*_S_ measurements, an AC excitation *δV*_EX_ is applied to both top and bottom gate. *δV*_S_ is then chosen to balance the bridge for a single set of DC voltages, i.e., such that *δv*_out_ = 0, in which case *C*_S_/*C*_std_ = *δV*_S_/*δV*_EX_. *n*_0_ and *p*_0_ are then swept and *δv*_out_ monitored, from which *C*_S_(*n*_0_, *p*_0_) is extracted. For *C*_A_, the measurement proceeds identically but with opposite phase signals applied to the two gates

Capacitance measurements were made using a cryogenic impedance transformer based on an FHX35X high electron mobility transistor^34^ in a bridge configuration connected to the bilayer graphene ohmic contacts (see Fig. 5). *v*_g_ sets the transistor bias point and *v*_d_ adjusted to be sufficiently low that no sample heating is observed. To measure *C*_S(A)_, two synchronized and nearly equal-magnitude AC signals (*δV*_EX_) are applied to the top and bottom gates, whose relative magnitude is chosen to match the ratio of geometric capacitances *c*_t_/*c*_b_ extracted from the DC characteristics of the device. The signals are applied in phase for *C*_S_ and out of phase for *C*_A_. A third AC signal is applied to a standard capacitor *C*_std_ with amplitude and phase that null the signal at the input of the cryogenic amplifier, and the capacitance and dissipation determined from the relation of the AC signals. *C*_std_ was measured to be 404 fF during the cooldown in which the data of Fig. 1 were measured. We used this value to determine *C*_S_ and *C*_A_ shown in subsequent figures, although in our experience *C*_std_ can vary by up to 20 fF from cooldown to cooldown, thus introducing a systematic uncertainty of approximately 5% in the capacitance shown in Figs. 2–4. All data shown are acquired off-balance, by monitoring the voltage at the balance point as DC values of the gate voltages are changed. Data in Fig. 2a and b are measured at 67.778 kHz using a 10 mV variation of *n*_0_/*c* and of *p*_0_/*c*, respectively.

Interpretation of *C*_A_ as a thermodynamic derivative requires that the sample is sufficiently conductive to fully charge over a time scale comparable to the inverse measurement frequency^35^. At low temperature and high magnetic fields, our sample becomes strongly insulating at integer filling factors, precluding this condition being satisfied. Failure to charge manifests as an increase in the out of phase, dissipative signal in the capacitance, allowing us to monitor charging across the parameter range. In Figs. 2b and 4a, regions in which the sample does not charge are masked in *black*, and dissipation data for all data sets is shown in Supplementary Figs. 1 and 2.

### Data availability

The data that support the findings of this study are available from the corresponding author upon reasonable request.

## Electronic supplementary material

Supplementary Information
