# Supplementary material for: Direct measurement of discrete valley and orbital quantum numbers in bilayer graphene
Source: Nat Commun. 2017 Oct 16;8:948. doi: 10.1038/s41467-017-00824-w (PMC5715057; doi:10.1038/s41467-017-00824-w)
Supplement: Supplementary file 1 — Supplementary Information [file 41467_2017_824_MOESM1_ESM.pdf]

Dissipation data associated with the data sets in the main text are shown in Supplementary Figs. 1 and 2. C_{A} data from a different device at high magnetic field is shown in Supplementary Fig. 3.

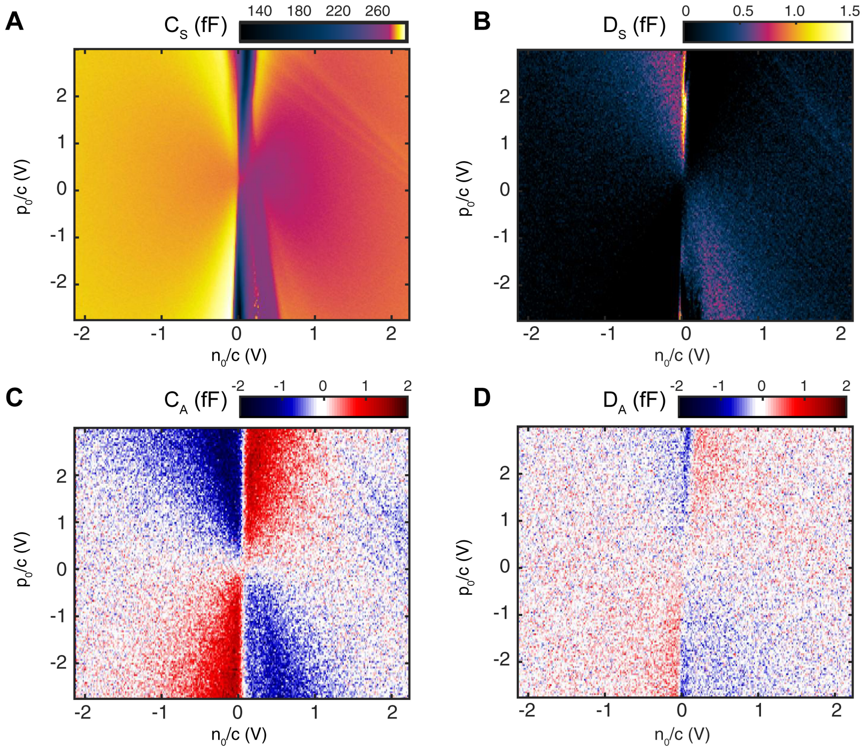

Supplementary Fig. 1. Capacitance and dissipation for the data set in Fig. 1 of the main text. Note that the scale in B is 1/100 of that in A.

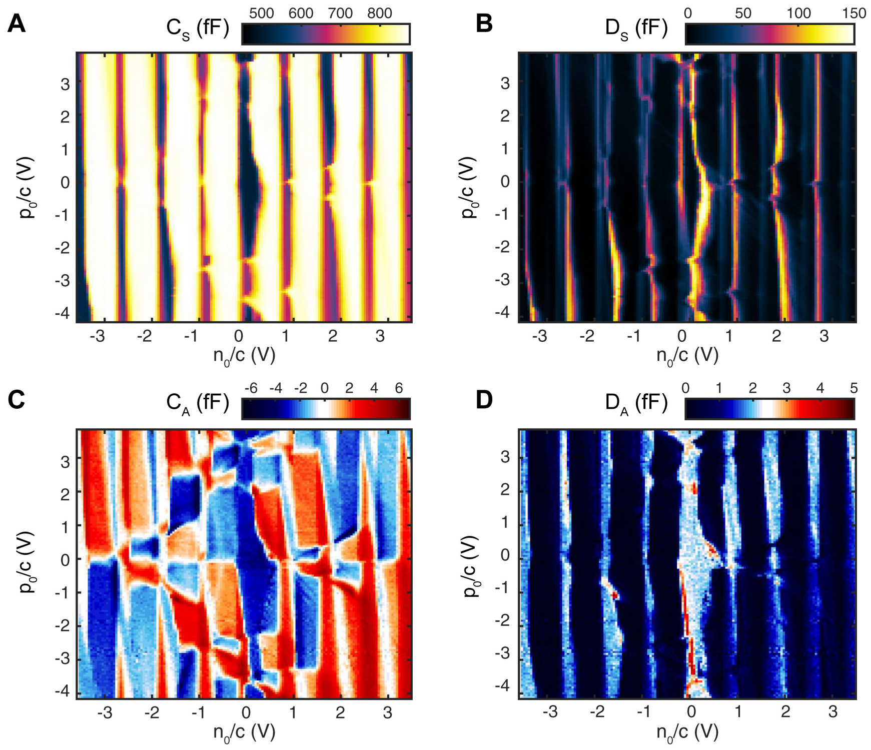

Supplementary Fig. 2. Capacitance and dissipation for the data set in Fig. 2 of the main text.

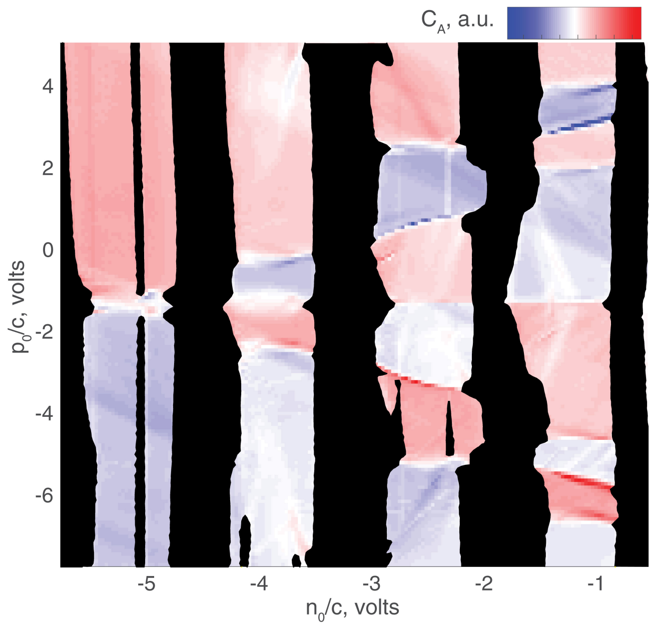

Supplementary Fig. 3. C_A measured in a different device at B=35T for $-4 < \nu < 0$. The device has nearly identical geometry to that described in the main text, but a larger area, enabling higher resolution measurements. Several fractional quantum Hall features are visible at one third filling of different LLs.

SUPPLEMENTARY NOTE 1: ELECTROSTATIC MODEL OF BILAYER GRAPHENE CAPACITANCE MEASUREMENTS

We model the dual gated graphene bilayer as a four plate capacitor, with the c_i corresponding to the geometric capacitances as indicated Fig. 1A of the main text. The n_i denote the areal *electron* densities on the four plates. Equations for the charge stored on each capacitor plate, as well as overall charge neutrality, result in four equations,

$$n_t + n_1 + n_2 + n_b = 0 \tag{1}$$
$$-c_{\rm t}(v_t - \phi_1) = n_t \tag{2}$$
$$-c_0(\phi_1 - \phi_2) = \frac{n_t + n_1 - n_2 - n_b}{2} \tag{3}$$
$$-c_{\rm b}(\phi_2 - v_{\rm b}) = -n_b. \tag{4}$$

Note that in the language of the main text, $\phi_2 - \phi_1 = u$. Suppl. Eqs. 1-4 are supplemented by the condition of electrochemical equilibrium between the top and bottom layers of the bilayer,

$$\phi_1 = v_0 - \mu_1 \tag{5}$$
$$\phi_2 = v_0 - \mu_2 \tag{6}$$

where v_0 is the voltage applied to the bilayer, and μ_i is the chemical potential on layer i. The μ_i depend on both n_1 and n_2 through the constitutive relations that derive from the electronic structure of the bilayer.

Capacitance measurements are performed with a small AC signal applied to one of three terminals while the corresponding variation in charge density is read out on another terminal. For small variations, then, the differential versions of Suppl. Eqs. 1-6 are relevant. In particular, Suppl. Eqs. 5,6 can be expressed in terms of the inverse compressibility matrix of the bilayer itself, $\kappa_{ij} = \partial\mu_i/\partial n_j$,

$$\delta\phi_1 = \delta v_0 - \kappa_{21}\delta n_1 - \kappa_{22}\delta n_2 \tag{7}$$
$$\delta\phi_2 = \delta v_0 - \kappa_{11}\delta n_1 - \kappa_{12}\delta n_2 \tag{8}$$

Note that $\kappa_{12} = \kappa_{21}$ follows from a Maxwell relation.

Experimentally, we measure the elements of the capacitance matrix

$$C_{ij}\left(\{c\},\{\kappa\}\right) = \left.\frac{\delta n_i}{\delta v_j}\right|_{\delta v_{k\neq j}=0}. \tag{9}$$

Where the indices indicate the voltages applied to the top gate (v_t), bottom gate ($v_{\rm b}$), or bilayer itself (v_0). Three elements of the capacitance matrix are independent, and we choose the most directly experimentally relevant combinations: the penetration field capacitance $C_{\rm P} \equiv -C_{\rm BT} = -C_{\rm TB}$, and top and bottom gate capacitances $C_{\rm B} \equiv C_{\rm B0}$ and $C_{\rm T} \equiv C_{\rm T0}$. Expressions for these three quantities can be found by varying Suppl. Eqs. 1-4 and using Suppl. Eqs. 7-8 to eliminate ϕ_1 and ϕ_2. All measurable capacitances depend on all three components of the compressibility matrix,

$$C_{\rm P} = \frac{c_{\rm b}c_{\rm t}(c_0\kappa_{11}\kappa_{22} - \kappa_{12} - c_0\kappa_{12}^2)}{1-(c_0+c_{\rm t})\kappa_{11} - (c_0+c_{\rm b})\kappa_{22} + (c_{\rm b}c_{\rm t} + c_0c_{\rm b} + c_0c_{\rm t})(\kappa_{11}\kappa_{22} - \kappa_{12}^2) + 2c_0\kappa_{12}} \tag{10}$$
$$C_{\rm B} = \frac{c_{\rm b}\left(1 + c_{\rm t}(\kappa_{11} - \kappa_12) + c_0(\kappa_{11} + \kappa_{22} - 2\kappa_{12})\right)}{1-(c_0+c_{\rm t})\kappa_{11} - (c_0+c_{\rm b})\kappa_{22} + (c_{\rm b}c_{\rm t} + c_0c_{\rm b} + c_0c_{\rm t})(\kappa_{11}\kappa_{22} - \kappa_{12}^2) + 2c_0\kappa_{12}} \tag{11}$$
$$C_{\rm T} = \frac{c_{\rm t}\left(1 + c_{\rm b}(\kappa_{22} - \kappa_12) + c_0(\kappa_{11} + \kappa_{22} - 2\kappa_{12})\right)}{1-(c_0+c_{\rm t})\kappa_{11} - (c_0+c_{\rm b})\kappa_{22} + (c_{\rm b}c_{\rm t} + c_0c_{\rm b} + c_0c_{\rm t})(\kappa_{11}\kappa_{22} - \kappa_{12}^2) + 2c_0\kappa_{12}}. \tag{12}$$

As described in the main text, the ultimate quantities of interest are the total density and layer density imbalance of the bilayer, $n \equiv n_1 + n_2$ and $p = n_1 - n_2$, while the most natural control parameters are $n_0 = c_{\rm t}v_t + c_{\rm b}v_{\rm b}$ and $p_0 = c_{\rm t}v_t - c_{\rm b}v_{\rm b}$. The partial derivatives of n with respect to n_0 and p_0 follow trivially from the fact that partial derivatives of the n with respect to the gate voltages can be measured directly:

$$\frac{\partial n}{\partial n_0} = \frac{\partial n}{\partial v_t}\frac{\partial v_t}{\partial n_0} + \frac{\partial n}{\partial v_{\rm b}}\frac{\partial v_{\rm b}}{\partial n_0} = \frac{1}{2}\left(\frac{C_{\rm T}}{c_{\rm t}} + \frac{C_{\rm B}}{c_{\rm b}}\right) = \frac{1}{2}\left(\frac{C_{\rm S}}{c}\right) \tag{13}$$
$$\frac{\partial n}{\partial p_0} = \frac{\partial n}{\partial v_t}\frac{\partial v_t}{\partial p_0} + \frac{\partial n}{\partial v_{\rm b}}\frac{\partial v_{\rm b}}{\partial p_0} = \frac{1}{2}\left(\frac{C_{\rm T}}{c_{\rm t}} - \frac{C_{\rm B}}{c_{\rm b}}\right) = \frac{1}{2}\left(\frac{C_{\rm A}}{c}\right). \tag{14}$$

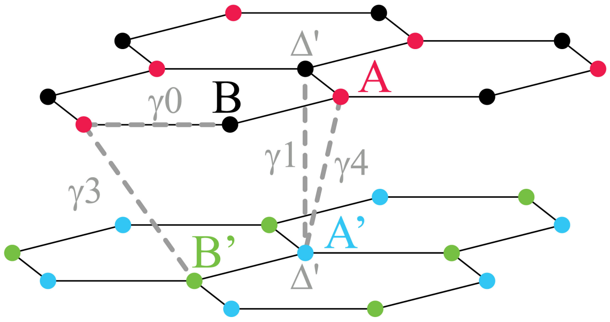

Supplementary Fig. 4. Bilayer graphene hopping parameters.

For convenience we have introduced the average geometric capacitance $c = \frac{c_b + c_t}{2}$ and the geometric capacitance asymmetry between top and bottom gates $\delta = \frac{c_b - c_t}{c_b + c_t}$; we also define here the capacitance observables measured and described in the main text as

$$C_{S(A)} \equiv \frac{C_B}{1-\delta} \pm \frac{C_B}{1+\delta}. \tag{15}$$

Derivatives of p can also be computed by varying 1-4 and using Suppl. Eqs. 7-8, to give expressions in terms of the κ_{ij}. After solving Suppl. Eqs. 10-12 for the three κ_{ij} and substituting the results, we arrive at expressions for these derivatives in terms of observable capacitances (C_P, C_S , and C_A):

$$\frac{\partial p}{\partial n_0} = \frac{\partial n_1}{\partial v_t}\frac{\partial v_t}{\partial n_0} + \frac{\partial n_1}{\partial v_b}\frac{\partial v_b}{\partial n_0} - \frac{\partial n_2}{\partial v_t}\frac{\partial v_t}{\partial n_0} - \frac{\partial n_2}{\partial v_b}\frac{\partial v_b}{\partial n_0} = \frac{1}{2}\left(\frac{\partial n_1}{\partial v_t}\frac{1}{c_t} + \frac{\partial n_1}{\partial v_b}\frac{1}{c_b} - \frac{\partial n_2}{\partial v_t}\frac{1}{c_t} - \frac{\partial n_2}{\partial v_b}\frac{1}{v_b}\right) \tag{16}$$

$$= \frac{1}{2}\left(\frac{c_t(\kappa_{11}+\kappa_{12}) + 2c_0(\kappa_{11}-\kappa_{22}) - c_b(\kappa_{12}+\kappa_{22})}{1-(c_0+c_t)\kappa_{11} - (c_0+c_b)\kappa_{22} + (c_b c_t + c_0 c_b + c_0 c_t)(\kappa_{11}\kappa_{22} - \kappa_{12}^2) + 2c_0\kappa_{12}}\right) \tag{17}$$

$$= -\frac{c_0}{c(1-\delta^2)}\left(\frac{C_A(1-\delta^2) + \delta((C_S - 2c)(1-\delta^2) + 4C_P)}{c(1-\delta^2)}\right) - \frac{4C_P\delta + C_A(1-\delta^2)}{c(1-\delta^2)} \tag{18}$$

$$\frac{\partial p}{\partial p_0} = \frac{\partial n_1}{\partial v_t}\frac{\partial v_t}{\partial n_0} - \frac{\partial n_1}{\partial v_b}\frac{\partial v_b}{\partial n_0} - \frac{\partial n_2}{\partial v_t}\frac{\partial v_t}{\partial n_0} + \frac{\partial n}{\partial v_b}\frac{\partial v_b}{\partial n_0} = \frac{1}{2}\left(\frac{\partial n_1}{\partial v_t}\frac{1}{c_t} + \frac{\partial n_1}{\partial v_b}\frac{1}{c_b} - \frac{\partial n_2}{\partial v_t}\frac{1}{c_t} - \frac{\partial n}{\partial v_b}\frac{1}{v_b}\right) \tag{19}$$

$$= \frac{1}{2}\left(\frac{2 + c_t(\kappa_{11}+\kappa_{12}) + c_b(\kappa_{12}+\kappa_{22})}{1-(c_0+c_t)\kappa_{11} - (c_0+c_b)\kappa_{22} + (c_b c_t + c_0 c_b + c_0 c_t)(\kappa_{11}\kappa_{22} - \kappa_{12}^2) + 2c_0\kappa_{12}}\right) \tag{20}$$

$$= \frac{c_0}{c(1-\delta^2)}\left(\frac{4C_P + (C_S - 2c)(1-\delta^2) + \delta C_A(1-\delta^2)}{c(1-\delta^2)}\right) - \frac{4C_P + C_S(1-\delta^2))}{2c(1-\delta^2)} \tag{21}$$

In our device the top and bottom gate geometric capacitances are nearly symmetric with $\delta \approx .029$; in addition, we estimate that $\frac{c}{2c_0} \lesssim .0086$. Taking the leading order terms in these two small parameters, we finally arrive at simplified expressions, some of which are used in the main text.

$$\frac{\partial n}{\partial n_0} \approx \frac{C_S}{2c} \tag{22}$$

$$\frac{\partial n}{\partial p_0} \approx -\frac{C_A}{2c} \tag{23}$$

$$\frac{\partial p}{\partial p_0} \approx \frac{c_0}{c}\frac{4C_P + C_S - 2c}{c} \tag{24}$$

$$\frac{\partial p}{\partial n_0} \approx -\frac{c_0}{c}\frac{C_A}{c} \tag{25}$$

SUPPLEMENTARY NOTE 2: THEORETICAL MODEL OF SYMMETRY BREAKING IN THE BLG ZLL

Outline

As described in the main text, calculating a phase diagram of bilayer graphene that correctly captures the experimentally observed effects requires considering a variety of both single particle and correlated electron effects. In this section we describe a minimal model which accounts for these effects, and compute ground state energies using Hartree-Fock and DMRG in order to determine the ν-dependence of the phase boundaries. The presentation is organized as follows: The Hamiltonian is a sum of the single-particle and two-body terms,

$$H = H^{(1)} + H^{(2)}. \tag{26}$$

We first describe the terms we choose to include in H. To make numerical progress, we then project the problem into the ZLL. Finally, in order to compute the energies of the competing phases at arbitrary ν, we perform both Hartree-Fock and DMRG computations.

Single-particle Hamiltonian

.

We start with a tight binding model of bilayer graphene that includes three intersite hopping terms (γ_0, γ_1, and γ_4 as well as the dimer on-site energy (Δ') but neglect the trigonal warping term (γ_3). A diagram of the bilayer graphene structure with relevant hopping integrals is shown in Fig. 4). At low energies, the Fermi surface has two disconnected parts near the corners of the Brillouin zone around the two inequivalent K points. The single particle Hamiltonian can then be reduced to a four-band model corresponding to the four sites of the BLG unit cell, resulting (in, e.g., valley K) in

$$\hat{H}_K^{B=0} = \begin{pmatrix} \frac{u}{2} & 0 & v_0\pi^\dagger & -v_4\pi^\dagger \\ 0 & -\frac{u}{2} & -v_4\pi & v_0\pi \\ v_0\pi & -v_4\pi^\dagger & \frac{u}{2}+\Delta' & \gamma_1 \\ -v_4\pi & v_0\pi^\dagger & \gamma_1 & -\frac{u}{2}+\Delta' \end{pmatrix} \qquad \begin{aligned} \gamma_0 &= -2.61\text{eV} \\ \gamma_1 &= .361\text{eV} \\ \gamma_4 &= .138\text{eV} \\ \Delta' &= .015\text{eV} \end{aligned} \tag{27}$$

where the basis in the K valley consists of the wavefunction weight on the four lattice sites in the bilayer graphene unit cell $(\phi_A, \phi_{B'}, \phi_B, \phi_{A'})$. Here $\pi = p_x - ip_y$ and $\pi^\dagger = p_x + ip_y$ are momentum operators, and $u = \phi_2 - \phi_1$ is the potential difference across the bilayer induced by the perpendicular electric field. Velocities are defined in terms of the monolayer graphene lattice constant, $a = 2.46$Å, as $v_0 = \frac{\sqrt{3}}{2}a\gamma_0/\hbar = 8.44 \times 10^5$m/s, $v_4 = \frac{\sqrt{3}}{2}a\gamma_4/\hbar = 4.47 \times 10^4$. We use values of the tight binding parameters from recent *ab inito* calculations[1] shown in Suppl. Eq. 27. Results for valley K' can be obtained by a 3D inversion, which exchanges $K \leftrightarrow K'$, $u \leftrightarrow -u$, $B \leftrightarrow A'$, and $B' \leftrightarrow A$.

To extend this Hamiltonian to the case of large perpendicular magnetic field, we introduce creation and annihilation operators for the scalar Landau level wavefunctions localized on each lattice site, defined as $\hat{a} \equiv \ell_B(q_x - iq_y)$ and $\hat{a}^\dagger \equiv \ell_B(q_x - iq_y)$ where $q_i \equiv k_i - \frac{e}{c}A_i$ and $\vec{A}$ is the magnetic vector potential. The operators operate on scalar Landau level wavefunctions such that $\hat{a}|n\rangle = \sqrt{n}|n-1\rangle$ and $\hat{a}^\dagger|n\rangle = \sqrt{n+1}|n+1\rangle$. The Hamiltonian in valley K, for example, then becomes

$$\hat{H}_K^B = \hbar\omega_0 \begin{pmatrix} \frac{u}{2\hbar\omega_0} & 0 & \hat{a}^\dagger & -\frac{\gamma_4}{\gamma_0}\hat{a}^\dagger \\ 0 & -\frac{u}{2\hbar\omega_0} & -\frac{\gamma_4}{\gamma_0}\hat{a} & a \\ \hat{a} & -\frac{\gamma_4}{\gamma_0}\hat{a}^\dagger & \frac{u}{2\hbar\omega_0} + \frac{\Delta'}{\hbar\omega_0} & \frac{\gamma_1}{\hbar\omega_0} \\ -\frac{\gamma_4}{\gamma_0}\hat{a} & \hat{a}^\dagger & \frac{\gamma_1}{\hbar\omega_0} & -\frac{u}{2\hbar\omega_0} + \frac{\Delta'}{\hbar\omega_0} \end{pmatrix} \tag{28}$$

where the monolayer graphene cyclotron energy is $\hbar\omega_0 = \frac{\hbar v_0\sqrt{2}}{\ell_B} \approx 30.6\sqrt{\text{B}_\perp/\text{Tesla}}$ meV. As at $B = 0$, the $H_{K'}$ follows from inversion, while spin enters only as an additional Zeeman energy σE_Z.

The eigenstates of Suppl. Eq. (28) take the general form

$$|\xi N\rangle = \sum_n (c^n_{\xi N;A}|n\rangle, c^n_{\xi N;B'}|n\rangle, c^n_{\xi N;B}|n\rangle, c^n_{\xi N;A'}|n\rangle), \tag{29}$$

where ξ labels the valley (henceforth denoted $\xi = \pm$), N labels the orbital quantum number, and the $|n\rangle$ are the oscillator states of $\hat{a}$, equivalent to the conventional quadratic-band LL-wavefunctions. The coefficients are then determined by the band-structure.

The $N \geq 2$ orbitals have energy $E_N \approx \hbar\omega_c\sqrt{N(N-1)}$, where $\hbar\omega_c \approx \frac{3a^2\gamma_0}{2\ell_B^2\gamma_1}\gamma_0$, while the $N = 0$ and 1 orbital are nearly degenerate, both having zero energy for $u = \gamma_4 = \Delta' = 0$. γ_4, Δ', and finite u all weakly lift this degeneracy, but still leave an eight fold near-degeneracy between states of different orbital, spin, and valley quantum numbers. The ZLL is well separated from the $N = \pm 2$ states at $E_{\pm 2} \approx \pm\sqrt{2}\hbar\omega_c \approx \pm 113$meV. While $N \geq 2$ LLs have support on all four sublattices in the unit cell, the $N = 0, 1$ eigenstates vanish on one or more sublattices:

$$|-0\rangle = (|0\rangle, 0, 0, 0) \tag{30}$$

$$|-1\rangle = (c_A|1\rangle, 0, c_B|0\rangle, c_{A'}|0\rangle)\,. \tag{31}$$

Defining the layer polarization as $\alpha \equiv |c_A|^2 - |c_{B'}|^2 + |c_B|^2 - |c_{A'}|^2$, we note that within the 4 band model the $N = 0$ orbital is fully layer polarized ($\alpha_0 = 1$) but the $N = 1$ is not; using the tight binding parameters above we find $\alpha_1 \approx .63$ at $B_\perp = 31$T. Wavefunctions in the opposite valley have correspondingly opposite layer polarization.

Throughout our experiment $u/\hbar\omega_c \ll 1$, and to leading order the single-particle energies of the ZLL are

$$H^{(1)} = -E_Z\sigma + \Delta_{10}N - \xi\frac{u}{2}\alpha_N. \tag{32}$$

Here, as in the main text, E_Z is the Zeeman energy, $\sigma = \pm\frac{1}{2}$ denotes the spin projection along the direction of the applied field, $\Delta_{10} \approx \hbar\omega_c(2\frac{\gamma_4}{\gamma_0} + \frac{\Delta'}{\gamma_1})$ is the single particle orbital splitting, $u = \phi_2 - \phi_1$ is the E-field induced potential difference across the bilayer, α_N is the layer polarization of the orbital, and $\xi = \pm$ indexes the valley. The single particle levels are shown in Fig. 3A of the main text. At $B = 31$T, $\Delta_{10} = 9.7$ meV, $E_Z = 3.58$ meV, and $\{\alpha_0, \alpha_1\} = \{1, 0.63\}$ as follows from Suppl. Eq. 28. The large splitting to the higher Landau levels ensures that they are not involved in any u- or E_Z-tuned phase transitions.

Coulomb Hamiltonian

The Coulomb interactions decompose into a dominant isospin SU(4)-symmetric part and subleading capacitive and valley anisotropies:

$$\mathbf{H}^{(2)} = \mathbf{H}^{\mathbf{SU(4)}} + \mathbf{H}^{c_0} + \mathbf{H}^{\mathbf{V}} \tag{33}$$

We now discuss these terms in turn.

$\mathbf{H}^{\mathbf{SU(4)}}$

Screened Coulomb interaction. The bare Coulomb interaction is screened by the surrounding hBN dielectric, the proximal metallic gates, and filled LLs below the ZLL of the BLG itself. The screening due to the hBN dielectric is incorporated as a dielectric constant in the Coulomb scale

$$E_C = \frac{e^2}{4\pi\epsilon_{\text{BN}}^{\parallel}\ell_B} \sim 8.58\sqrt{\text{B/T}}\text{meV}, \tag{34}$$

assuming $\epsilon_{\text{BN}}^{\parallel} \approx 6.6\epsilon_0$[2,3]. The metallic gates, each at distance $D \approx 20$nm from the bilayer, exponentially screen the interaction when $r \gg D$. The 2D Fourier transform of the gate-screened potential is

$$V(k) = \frac{2\pi}{k}\tanh(kD) \tag{35}$$

in units of E_C and ℓ_B. Here we neglect the finite width of the bilayer itself, which is an order of magnitude smaller than ℓ_B; it will be reincorporated as a capacitive energy below.

Since we wish to work with a model projected into the ZLL, we must account for the residual response of the other LLs, colloquially referred to as "LL-mixing". The dimensionless parameter controlling their response is $\frac{E_C}{\hbar\omega_0} \sim 3.14/\sqrt{B/\text{T}} \approx 0.56$ at $B = 31$T, which is comparable to values in GaAs. Motivated by the large number of isospin flavors (four), the standard approach for BLG is the random phase approximation (RPA) ,[4,5] in which we replace the bare Coulomb potential $V(k)$ with the effective potential

$$V_{\text{eff}}(\omega, k) = \frac{V(k)}{1 + V(k)\Pi(\omega, k)}, \tag{36}$$

where Π is the polarization response. The RPA result is then further approximated by the static $\omega = 0$ value. However, RPA calculations have only been reported for the PH-symmetric two-band model[5] at $\nu = 0$, which is not quantitatively correct at large electric fields or at the magnetic fields relevant for our experiment[6]. Moreover, Ref. 5 found that the static $\omega = 0$ approximation strongly overestimates screening (IQHE gaps were underestimated by a factor of three). Thus even recalculating the RPA value with a four band model is unlikely to be quantitatively accurate, as it is not possible to incorporate ω dependence into ground state numerical methods like exact diagonalization or DMRG. Thus, at present, there does not appear to be a satisfactory "ab initio" tool for quantitatively predicting the strength of screening.

For these reasons, we use a phenomenological model following the approach of Ref. 7, taking

$$V_{\text{eff}}(k) = \frac{V(k)}{1 + aV(k)\tanh(bk^2\ell_B^2)4\log(4)/2\pi} \tag{37}$$

This is motivated by the RPA form when approximating the polarization as $\Pi(k) = a\tanh(bk^2\ell_B^2)4\log(4)/2\pi$, with $V(k)$ given in Suppl. Eq.(35). If a, b are chosen to match the low-k and high-k behavior of the $\nu = 0$ two-band RPA calculation,[5] one finds $a_{\text{RPA}} \equiv \frac{E_C}{\hbar\omega_0}$ and $b_{\text{RPA}} \equiv 0.62$. However, much more generally we must have $\Pi(k) \propto k^2$ at low-k and $\Pi(k) \to$ const at high-k, as captured by the ansatz. Quantum Hall calculations are only sensitive to the form of the interaction in the vicinity of $k \lesssim \ell_B^{-1}$, so the magnitude of the low-k behavior (k^2) forms a one-parameter space of screening behaviors set here by the product ab. Thus we fix $b = b_{\text{RPA}}$, and treat a_{scr} as a phenomenological measure of the screening strength.

ZLL projected Hamiltonian– We then project the screened Coulomb interaction $V_{\text{eff}}(q)$ into the eight components of the ZLL. For the moment we neglect the small, lattice-scale valley anisotropies which break the valley-SU(2) symmetry; these effects will be introduced as phenomenological couplings shortly. The SU(4)-isospin symmetric interaction is

$$\mathbf{H}^{\mathbf{SU(4)}} = \tfrac{1}{2}\int \frac{d^2q}{(2\pi)^2}\, n_{\text{ZLL}}(q)V_{\text{eff}}(q)n_{\text{ZLL}}(-q) + \Delta_{\text{Lamb}}\sum_{\sigma\xi}\hat{N}_{\xi 1\sigma} \tag{38}$$

Here $n_{\text{ZLL}}(q) = \sum_{\xi\sigma} n_{\xi\sigma}(q)$ is the total density in the ZLL, which is a sum of the four isospin components, while $\hat{N}_{\xi 1\sigma}$ is the electron number in level $\xi 1\sigma$. As explained in Ref. 8, a shift Δ_{Lamb} between the $N = 0, 1$ orbitals arises when projecting into the ZLL, since their Coulomb exchange with the filled LLs below the ZLL differs. Under the approximation of particle-hole symmetry, it was shown that $\Delta_{\text{Lamb}} = \frac{1}{2}(E_{00}^{(\text{ex})} - E_{11}^{(\text{ex})}) < 0$, where $\frac{1}{2}E_{NN}^{(\text{ex})}$ is the Coulomb exchange per-electron when fully filling an N level with a Slater-determinant. We evaluate this shift using the screened interaction V_{eff}, and find a near perfect fit to $\Delta_{\text{Lamb}} \simeq -\frac{0.2E_C}{1+2.73a_{\text{scr}}}$.

The density $n_{\xi,\sigma}(q)$ contains a contribution from both the $N = 0$ and $N = 1$ levels. The ratio between the Coulomb scale and the single-particle splitting between the orbitals is

$$\frac{E_C}{\Delta_{10} + \Delta_{\text{Lamb}} + \xi\frac{u}{2}(\alpha_0 - \alpha_1))} \approx \frac{E_C}{\Delta_{10} + \Delta_{\text{Lamb}}} \approx 12. \tag{39}$$

Thus, unlike the levels outside the ZLL, it is not well-justified to project the interaction into only one of the two N orbitals; we must keep both.

BLG form factors– For completeness, we explain how the BLG "form factors" $\mathcal{F}_{NM}$ can be used to compute the Coulomb matrix elements projected into the Landau-level basis, which are required for the actual computations. The density $n_{\sigma\xi}$ is not diagonal in the orbital index N, but instead involves "orbital-mixing" contributions:

$$n_{\sigma\xi}(q) = \sum_{N,M=0,1} \bar{\rho}_{\sigma\xi NM}(q)\mathcal{F}_{NM}(q) \tag{40}$$

Here $\bar{\rho}$ is a guiding-center density operator, which in the Landau gauge reads

$$\bar{\rho}_{\sigma\xi NM}(q) \equiv \sum_k e^{-ikq_x\ell_B^2}\psi^{\dagger}_{\sigma\xi N}(k + q_y/2)\psi_{\sigma\xi M}(k - q_y/2). \tag{41}$$

The form factor $\mathcal{F}$ is expressed in terms of the conventional quadratic-band form factors F_{nm} as

$$\mathcal{F}_{NM}(q) = \sum_{A_i,m,n} \bar{c}^{n}_{\xi N;A_i} F_{nm}(q) c^{m}_{\xi M;A_i}. \tag{42}$$

where $A_i = A, B, A', B'$ are the sublattices and c are the wavefunction amplitudes defined in Suppl. Eq.(29). The $\mathcal{F}_{NM}$ are independent of ξ, since to leading order in u the $\xi = \pm$ wavefunctions differ only by a permutation of the sites.

In the four-band model, we refer to Suppl. Eqs.(30)-(31) to find

$$\mathcal{F}_{00} = F_{00}, \quad \mathcal{F}_{01} = c_A F_{01}, \quad \mathcal{F}_{11} = c_A^2 F_{11} + (c_{A'}^2 + c_B^2) F_{00}. \tag{43}$$

It is thus convenient to parameterize the interaction by $\cos^2\Theta = c_A^2, \sin^2\Theta = c_{A'}^2 + c_B^2$, where $\Theta \approx 0.44$ at B =31T. At low perpendicular magnetic fields, $\Theta \to 0$ and the problem reduces to the two-band model

$$\mathcal{F}_{00} = F_{00}, \quad \mathcal{F}_{01} = F_{01}, \quad \mathcal{F}_{11} = F_{11}, \tag{44}$$

equivalent to the two lowest Landau-levels of the conventional quadratic-band QHE.

$\mathbf{H}^{c_0}$ *and* $\mathbf{H}^{\mathbf{V}}$

Lattice scale effects at order a/ℓ_B generate valley-SU(2) breaking perturbations sensitive to the details of the lattice and orbital structure, so must be treated phenomenologically[9,10]. The first anisotropy is a capacitive energy due to the finite thickness of the BLG (d), which modifies the interlayer Coulomb interaction from $V(q) \to V(q)e^{-qd}$. This perturbation is smaller than the long range Coulomb ($H^{SU(4)}$) by a factor of $d/\ell_B \ll 1$, but the $q = 0$ part is unscreened, so for simplicity we retain the Hartree-type capacitive charging energy $E^{c_0} = \frac{1}{8c_0}(N_t - N_b)^2$, where $N_{t/b}$ is the charge on the top / bottom layer, evaluated using α_1. Writing the capacitance of the BLG as $c_0 = \frac{\epsilon^{\perp}_{\mathrm{BLG}A}}{d}$, we can manipulate the expression to obtain

$$\mathbf{H}^{c_0} = N_\Phi E_C \frac{d}{\ell_B} \frac{\epsilon^{\parallel}_{\mathrm{BN}}}{\epsilon^{\perp}_{\mathrm{BLG}}} \frac{(\nu_t - \nu_b)^2}{4}. \tag{45}$$

The ratio of dielectric constants arises because we included $\epsilon^{\parallel}_{\mathrm{BN}}$ in E_C. The dielectric constant $\epsilon^{\perp}_{\mathrm{BLG}}$ should be the same one used for converting the applied gate voltage p_0/c to the inter-layer bias u.

Following Refs. 9 and 10, the remaining valley anisotropies are incorporated as short-range (δ-function) interactions which preserve spin $SO(3)$ and valley $U(1) \times U(1) \rtimes \mathbb{Z}_2$. In contrast to monolayer graphene, symmetry considerations allow a rather large space of possible perturbations since they can take an arbitrary form in the $N = 0, 1$ orbital index. Here we will assume the perturbations can be expressed using local operators that depend only on the isospin, e.g. $O^{\mu\nu}(r) = \psi^\dagger_{\xi,\sigma}(r)\tau^\mu_{\xi,\xi'}\sigma^\nu_{\sigma,\sigma'}\psi_{\xi',\sigma'}(r)$, where $\psi_{\xi,\sigma} = \sum_N \psi_{\xi,N,\sigma}(r)$ and τ, σ are Pauli operators. This assumption is reasonable when $\Theta \to 0$, since the anisotropies arise from lattice structure and the $N = 0, 1$ orbitals sit on the same site in this limit. While this approximation neglects the $\Theta \neq 0$ corrections due to the finite polarization of the N=1 levels, we note that the capacitance term $\mathbf{H}^{c_0}$ captures at least some of these.

We express the anistropy energy using the total valley number density $n_\pm(r)$ and the valley spin density $\mathbf{S}_\pm(r) = \frac{1}{2}\sum_{MNab}\psi^\dagger_{\pm Ma}(r)\sigma_{ab}\psi_{\pm Nb}(r)$. In addition to the total density (which preserves SU(4)), the most general symmetry preserving perturbation is

$$\mathbf{H}^{\mathbf{V}} = E_C \frac{d}{\ell_B} 2\pi\ell_B^2 \int d^2r \left[-g_z n_+(r) n_-(r) + g_\perp \mathbf{S}_+(r)\cdot\mathbf{S}_-(r)\right] \tag{46}$$

Note that previous treatments[9] have assumed interactions of the type $\frac{u_\perp}{2}(\tau^x\tau^x + \tau^y\tau^y)$ and $\frac{u_z}{2}\tau^z\tau^z$. These forms are in fact equivalent: using the anti-symmetry of the fermions, we have $E_C\frac{d}{\ell_B}g_\perp = -4u_\perp, E_C\frac{d}{\ell_B}g_z = u_\perp + 2u_z$.

Evaluation of $H^{(2)}$

Our model depends on a number of parameters, summarized in Tab. I. Some are known from the literature (e.g., $\epsilon^{\parallel}_{\mathrm{BN}}$ and the tight binding parameters shown in Suppl. Eq. 27), some can be derived directly from these assumed values (e.g., $\Delta_{10}, \alpha_1, E_C$), and some follow from theory (e.g., $\zeta, \Delta_{\mathrm{Lamb}}$). In addition, there are several phenomenological parameters which we constrain from experiment, namely $\epsilon^{\perp}_{\mathrm{BN}}, g_\perp, g_z, a_{\mathrm{scr}}, \epsilon^{\perp}_{\mathrm{BLG}}$.

To get a handle on these parameters, we begin with a Hartree Fock approximation for $H^{(2)}$, which allows us to conveniently estimate the predicted location of the transitions within our model.

TABLE I. Model parameters

Parameter	Expression	Value	Source
$\epsilon_{BN}^{\parallel}$	–	6.6 ϵ_0	literature[2,3]
$\epsilon_{BN}^{\perp}$	–	3.0 ϵ_0	measured (Sec.)
Δ_{10}	$E_{\xi 1\sigma}(u=0) - E_{\xi 0\sigma}(u=0)$	9.7 meV	derived from band structure
α_1	$\phi_A^2 - \phi_{B'}^2 + \phi_B^2 - \phi_{A'}$	0.63	derived from band structure[1]
$\hbar\omega_c$	$\frac{3a^2\gamma_0}{2\ell_B^2\gamma_1}\gamma_0$	80 meV	derived from band structure[1]
E_C	$\frac{e^2}{4\pi\epsilon_{BN}^{\parallel}\ell_B}$	47.3 meV	derived from $\epsilon_{BN}^{\parallel}$
a_{RPA}	$\frac{E_C}{\hbar\omega_c}$	.42	derived from E_C and $\hbar\omega_c$
ζ	$\frac{E_C}{2}(-E_{00}^{(\text{ex})} + E_{11}^{(\text{ex})} + 2E_{01}^{(\text{ex})}) = -\frac{0.315E_C}{1+2.52a_{\text{scr}}}$	-8.8 meV	calculated (Suppl. Eq. 48)
Δ_{Lamb}	$\frac{E_C}{2}(E_{00}^{(\text{ex})} - E_{11}^{(\text{ex})}) = -\frac{0.2E_C}{1+2.73a_{\text{scr}}}$	-5.4 meV	calculated (Suppl. Eq. 49)
$g_\perp$	–	0.69	estimated from data (Sec.)
g_z	–	0.45	fit from phase transitions (Sec.)
$\epsilon_{BLG}^{\perp}$	–	2.8	fit from phase transitions (Sec.)
a_{scr}	–	.28≈.67a_{RPA}	fit from phase transitions (Sec.)

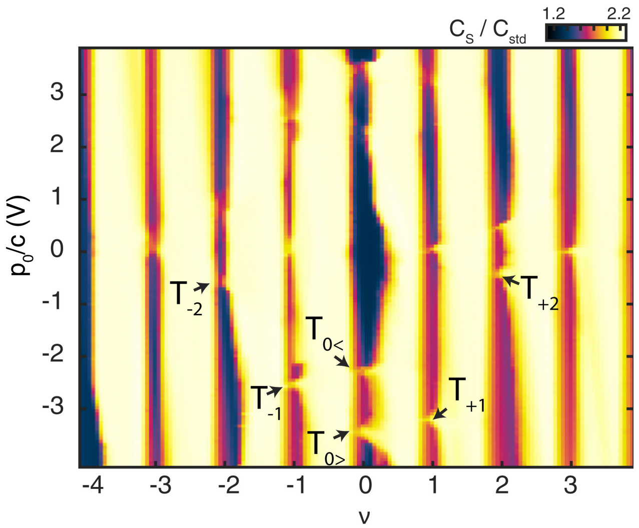

Supplementary Fig. 5. C_{S} with phase transitions labeled for reference.

Hartree Fock approximation for $H^{(2)}$

Within the Hartree-Fock approach, we assume that the ground-state at integer filling is a Slater determinant which successively fills orbitals $\xi N\sigma$ according to the proposed phase. We neglect the possibility of valley off-diagonal coherence (e.g., non-zero $\langle\psi_\xi^\dagger\psi_{\xi'}\rangle \neq 0, \xi \neq \xi'$). While such phases have been predicted to occur, for instance in a very narrow range of p_0 at $\nu = -3$,[11] there is no evidence for them in the current data, since there appears to be a single direct transition at $p_0 = 0$.

The energy of $\mathbf{H}^{\mathbf{SU(4)}}$ at integer filling can then computed as an integral involving $\mathcal{F}_{NM}$ and V_{eff} (see, for example, Ref. 11). In contrast to earlier works, we use both the screened interactions and the form factors appropriate to the $B = 31$T four-band model, as described above. We interpolate the integer result to fractional ν by generalizing the first term in the interpolation of Fano and Ortolani[12] to the multi-component case:

$$\frac{E^{SU(4)}}{N_\Phi} \approx \frac{E_C}{2}\sum_{\sigma\xi N,N'} \nu_{\sigma\xi N} E_{N,N'}^{(\text{ex})} \nu_{\sigma\xi N'} + \Delta_{\text{Lamb}} \sum_{\sigma\xi} \nu_{\sigma\xi 1} \tag{47}$$

The entry $\frac{1}{2}E_{00}^{(\text{ex})}$ is the energy to fill a $N = 0$ level; $\frac{1}{2}E_{11}^{(\text{ex})}$ the energy to fill a $N = 1$ level; and $\frac{1}{2}E_{00}^{(\text{ex})} + \frac{1}{2}E_{11}^{(\text{ex})} + E_{01}^{(\text{ex})}$ is the

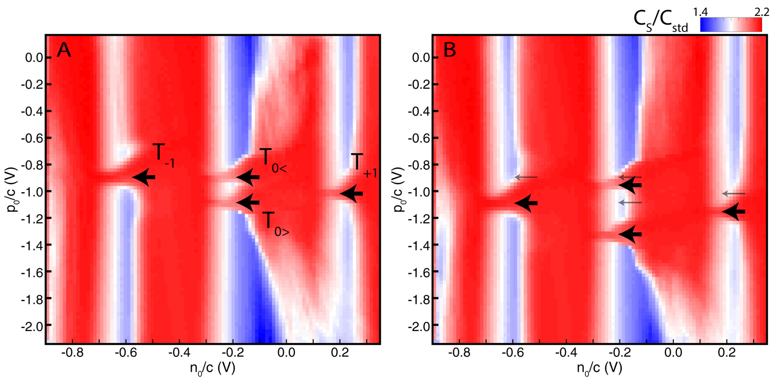

Supplementary Fig. 6. Tilted magnetic field dependence of integer transitions. (**a**) Finite p_0 transitions at $\nu = 0$ and $\nu = \pm 1$ at $B_{tot} = B_\perp = 15T$. (**b**) Similar data at $B_{tot} = 31T$ and $B_\perp = 15T$. Arrows indicate tilted and non-tilted positions of the phase transitions. The shifts are listed in Tab. II

energy to fill both. The splitting between filling $N = 0, 1$ orbitals of the same isospin relative to two $N = 0$ orbitals of opposite isospin is then $\zeta = \frac{E_C}{2}(-E^{(\text{ex})}_{00} + E^{(\text{ex})}_{11} + 2E^{(\text{ex})}_{01})$. Calculations were repeated for a range of a_{scr}, and we find

$$\zeta = -\frac{0.315 E_C}{1 + 2.52 a_{\text{scr}}} \tag{48}$$

gives an almost perfect interpolation of the result. The Lamb shift, meanwhile, is found to be

$$\Delta_{\text{Lamb}} = \frac{E_C}{2}(E^{(\text{ex})}_{00} - E^{(\text{ex})}_{11}) = -\frac{0.2 E_C}{1 + 2.73 a_{\text{scr}}}. \tag{49}$$

Note that this expression for the Lamb shift remains true beyond Hartree-Fock, so will also be used in our DMRG calculation.

The valley-anisotropies are off-diagonal in the valley index, so they reduce to a Hartree energy, e.g. $\langle n_+(r) n_-(r)\rangle \to \langle n_+(r)\rangle\langle n_-(r)\rangle$. Using $n_\pm(r) = \frac{1}{2\pi \ell_B^2}\nu_\pm$, we arrive at the final expression

$$\frac{E^{(2)}_{\text{H.F.}}}{E_C} = \frac{1}{2}\sum_{\xi N\sigma, N'} \nu_{\sigma\xi N} E^{(\text{ex})}_{N,N'} \nu_{\sigma\xi N'} + \Delta_{\text{Lamb}} \sum_{\sigma\xi} \nu_{\sigma\xi 1} + \frac{1}{4}\frac{d}{\ell_B}\frac{\epsilon^{\parallel}_{\text{BN}}}{\epsilon^{\perp}_{\text{BLG}}}(\nu_t - \nu_b)^2 - g_z \frac{d}{\ell_B}\nu_+\nu_- + g_\perp \frac{d}{\ell_B}\mathbf{S}_+\cdot\mathbf{S}_- \tag{50}$$

Experimental determination of $\epsilon^{\perp}_{BN}$

Since $\epsilon^{\perp}_{\text{BN}}$ determines the capacitance between the gates and BLG, we can measure $\epsilon^{\perp}_{\text{BN}}$ by fitting the Landau level spacing, as measured in the applied gate voltages n_0/c, to their known densities $n = \frac{\nu}{2\pi\ell_B^2}$. Data were taken at $p_0 = 0$ and $B_\perp = 2T$. Starting from the electrostatic model of Suppl. Eqs. 1-2, we ignore interlayer capacitance ($c_0 \sim \infty$) to treat the bilayer as a single 2D electron system, and neglect the finite quantum capacitance, which is reasonable at low fields. Fitting the separation, in n_0, of two four fold degenerate LLs to $\Delta n_0 = \epsilon^{\perp}_{\text{BN}}/d_{BN}\,(\Delta v_t + \Delta v_{\text{b}}) = \frac{4}{2\pi\ell_B^2}$, we find $\epsilon^{\perp}_{\text{BN}} \sim 3.0 \pm .15$.

Experimental estimate of $g_\perp$

By measuring the critical total B-field of the ferromagnet to canted antiferromagnetic transition at $\nu = p_0 = 0$, previous experiments[13] have estimated that $B^{\text{tot}}_* = (2.42 \pm 0.21)B^\perp$. This implies $u_\perp = 0.14$meV/T, or in our parameterization, $g_\perp = \frac{u_\perp}{E_C}\frac{\ell_B}{d} = 1.2$. While this experiment shows the expected $B^\perp$-linear scaling for $B^\perp = 4 - 7$ T, we must be careful when extrapolating to higher magnetic fields.

To address this question, we analyze the tilted field measurements of four phase transitions at $B_\perp = 15$T, as shown in Fig. 6. From the experimental data, we extract the shift in $\Delta p_0/c$ over the probed magnetic field range. Each transition is expected to

Transition	$\Delta(p_0/c)$, V	$\overline{\alpha}$	ϵ_{blg}^{min}	$\overline{\sigma}$, $\frac{\epsilon_{blg}}{\epsilon_{BN}^{\perp}} = \frac{2.76}{3}$
T_{-1}	.21(.025)	1.0	2.58	1.06 (.13)
$T_{0<}$	.23(.025)	0.9	0.31	.23 (.11)
$T_{0>}$	.05(.025)	0.9	2.57	1.05 (.11)
T_{+1}	.13(.025)	0.8	1.17	.53 (.1)

TABLE II. Effective spin involved in phase transitions near $\nu = 0$, extracted from the data shown in Fig. 6 and Suppl. Eq. 51

ν	Transition	Canting?	Constraint
-2	T_{-2}	no	$E_Z > g_\perp E_c \frac{d}{\ell_B}$
-1	T_{-1}	no	$E_Z > \frac{3}{2} g_\perp E_c \frac{d}{\ell_B}$
0	$T_{0<}$	yes	$E_Z < 2 g_\perp E_c \frac{d}{\ell_B}$
0	$T_{0>}$	no	$E_Z > g_\perp E_c \frac{d}{\ell_B}$
+1	T_{+1}	yes	$E_Z < \frac{3}{2} g_\perp E_c \frac{d}{\ell_B}$

TABLE III. Constraints on $g_\perp$ implicit in the presence of absence of spin canting at five phase transitions. Transitions refer to Fig. 6. No tilted field dependence is ever observed at $\nu = -2$, consistent with full spin polarization.

shift by $\Delta u = \frac{\overline{\sigma}\Delta E_Z}{\overline{\alpha}}$, where $\overline{\alpha}$ is the effective change in layer polarization across the transition and $\overline{\sigma}$ is the effective change in spin, and $\Delta E_Z = 0.116 \Delta B^{\rm tot}$ meV/T the change in Zeeman energy. Solving for $\overline{\sigma}$, we find

$$\overline{\sigma} = \frac{c}{2c_0}\Delta(p_0/c)\overline{\alpha} = .0086 \frac{\epsilon_{\rm BN}^{\perp}}{\epsilon_{\rm BLG}^{\perp}} \frac{\Delta(p_0/c)}{\Delta E_Z}\overline{\alpha}, \tag{51}$$

where $0.0086 = \frac{0.335\rm nm}{39\rm nm}$ is the ratio of geometric capacitances. When calculating $\bar{\alpha}$, we must remember that $\alpha_1 = 0.8$ at $B_\perp$=15T, as extracted from band structure parameters[1]. In the absence of antiferromagnetism, all transitions considered involve reversal of one full electron spin, so that $\overline{\sigma} = 1$. Canting of the spins due to $g_\perp$ can reduce the effective spin; however, $\overline{\sigma} > 1$ is unphysical as it would imply a larger than unity spin per electron. Taking into account experimental error, each tilted field data point thus imposes a lower limit on $\epsilon_{\rm BLG}^{\perp}$, the most stringent of which is $\epsilon_{\rm BLG}^{\perp} > 2.57$. As we will see below, this limit is ultimately consistent and does not influence the final fitted value of $\epsilon_{\rm BLG}^{\perp} = 2.76$.

Using this value for $\epsilon_{\rm BLG}^{\perp}$, we can then calculate $\overline{\sigma}$ for each transition, shown in Table II. Two of the four spin transitions are consistent with absence of canting ($\overline{\sigma} = 1$), and two are consistent with strong canting ($\overline{\sigma} < 1$). Additional data (not shown) shows that the phase transitions at $\nu = -2$ do not depend on in-plane magnetic field, suggesting that antiferromagnetism has not yet set in at this filling.

In a phase characterized by net spin S_+ and S_- in each valley, the threshold for canting in our model arises from the interplay between the Zeeman effect (which favors polarization) and $g_\perp$ (which favors canting) via

$$E_Z^c = g_\perp E_c \frac{d}{\ell_B}(S_+ + S_-), \tag{52}$$

where observation of $\overline{\sigma} < 1$ at a phase transition implies that $E_Z < E_Z^c$ for one of the adjacent phases. The canting at $T_{0<}$ and the absence of canting at both T_{-2} and $T_{0>}$ together constrain $0.52 < g_\perp < 1.04$. T_{-1} and T_{+1}, meanwhile, seem to give contradictory constraints, $0.69 < g_\perp$ and $0.69 > g_\perp$. The lack of consistency likely stems from the dependence of the $g_\perp$ anisotropy on the orbital filling: T_{-1} and T_{+1} involve different partial fillings of an isospin flavor, and $g_\perp$ need not be identical for different orbital combinations in the different valleys. At T_{-1}, occupation is transferred from a $|0+\uparrow\rangle$ to a $|0-\downarrow\rangle$ state, while at T_{-1} occupation is transferred from a $|1+\sigma\rangle$ to a $|1-\sigma'\rangle$ state. As we discussed, this effect is not captured in Suppl. Eq. 46, which treats orbital components equally. While the precise value and possible orbital substructure of $g_\perp$ is critical for determining the precise dependence of the antiferromagnetism on filling, within our model we find $g_\perp$ has negligible impact on the locations of the u-tuned phase transitions, which are the focus of this work. Thus we set $g_\perp = 0.69$, and defer a more full analysis of the ν dependence of the antiferromagnetism to future high resolution studies of the tilted field dependence of the phase transitions observed in $C_{\rm A}$.

Name	meas. value (error)	best fit	unit
T_{-2}	0.68 (.04)	0.63	V
T_{-1}	2.59 (.05)	2.57	V
$T_{0>}$	3.48 (.05)	3.6	V
T_{+1}	3.27 (.05)	3.2	V
$\partial V(T_{-1})/\partial B^{\text{tot}}$	13 (2.5)	12.7	mV/T

TABLE IV. Experimentally measured constraints.

Estimating $g_z, a_{scr}, \epsilon^{\perp}_{BLG}$ from the experimental data

In order to estimate $g_z, a_{\text{scr}}, \epsilon^{\perp}_{\text{BLG}}$, we compare the Hartree-Fock model with experiment. Given the Hartree-Fock energy $E^{(\text{HF})}_{\nu_{\xi N\sigma}} = \langle H^{(1)} + H^{(2)} \rangle_{\nu_{\xi N\sigma}}$ of a filling sequence, the phase boundaries $u_*(\nu)$ are determined by equating $E^{(\text{HF})}_{\nu_{\xi N\sigma}} = E^{(\text{HF})}_{\nu'_{\xi N\sigma}}$. The layer bias u_* is then related to the experimentally measured gate bias via $\frac{p_0}{c} = u\frac{2c_0}{c} = \frac{\epsilon^{\perp}_{\text{BLG}}}{\epsilon^{\perp}_{\text{BN}}}\frac{40\text{nm}}{0.335\text{nm}}$. Experimental data points are provided by the positions, in p_0/c, of the transitions T_{-2}, T_{-1}, $T_{0>}$, and T_{+1} (see Fig. 5 for the labeling scheme). In the absence of canting (we have assumed $g_\perp = 0.69$), the predicted phase boundaries are

$$u_*(T_{-2}) = \frac{2}{1+\alpha_1}\left(\zeta + \Delta_{\text{Lamb}} + \Delta_{10} + \frac{E_C}{4\epsilon^{\perp}_{\text{BLG}}}\frac{d}{\ell_B}(1+\alpha_1)^2 - E_C\frac{d}{\ell_B}\left[-g_z + g_\perp\frac{1}{4}\right]\right) \tag{53}$$

$$u_*(T_{-1}) = \left(\frac{E_C}{\epsilon^{\perp}_{\text{BLG}}}\frac{d}{\ell_B}[1+\alpha_1] - E_C\frac{d}{\ell_B}\left[-2g_z + \frac{1}{2}g_\perp\right] + E_Z\right) \tag{54}$$

$$u_*(T_{0>}) = \frac{2}{1+\alpha_1}\left(\zeta + \Delta_{\text{Lamb}} + \Delta_{10} + \frac{E_C}{4\epsilon^{\perp}_{\text{BLG}}}\frac{d}{\ell_B}3(1+\alpha_1)^2 - E_C\frac{d}{\ell_B}\left[-3g_z + g_\perp\frac{1}{4}\right] + E_Z\right) \tag{55}$$

$$u_*(T_{+1}) = \frac{1}{\alpha_1}\left(\frac{E_C}{4\epsilon^{\perp}_{\text{BLG}}}\frac{d}{\ell_B}\left[(1+2\alpha_1)^2 - 1\right] + E_C\frac{d}{\ell_B}\left[2g_z - \frac{1}{2}g_\perp\right] + E_Z\right) \tag{56}$$

To compare to experimental values of p_0/c for each transition, we convert $V(T_i) = u_*(T_i)\frac{2c_0}{c}$. An fifth constraint arises from the tilt-field dependence of Suppl. Eq. (54), which implies

$$\frac{\partial V(T_{-1})}{\partial B^{\text{tot}}} = \frac{2c_0}{c}g\mu_B \approx .00462\epsilon^{\perp}_{\text{BLG}}\frac{\text{meV}}{\text{T}} \tag{57}$$

Given five experimental constraints in three unknowns, we do a least-squares fit for our overconstrained model. The model is validated, simultaneously fitting all data points across a wide range of ν, and providing physically reasonable values for $\epsilon^{\perp}_{\text{BLG}} = 2.76$, $a_{\text{scr}} = 0.29 = 0.5a_{\text{RPA}}$, and $g_z = .082$. The resulting phase diagram is plotted in Fig. 3c of the main text, lower panel, and similarly shows good quantitative agreement with most of the features of the experimentally measured phase diagram.

Phase boundaries from iDMRG

In order to reproduce the kink in $u_*(\nu)$ observed in experiment around $-3 < \nu < -1$, we replace the Fano-Ortolani interpolation of the HF result (Suppl. Eq. (47)) with the infinite-DMRG calculation, which takes full account of correlations. As before, we must compute the energies of the competing phases. Since the tilt-field dependence shows that the spins are polarized across the transition, we label the isospin only by its valley $\xi = \pm$. At the transition, density is transferred between isospin components. The cartoon picture is that in phase "001" (low p_0), the orbitals fill in order $+0, -0, +1$, while in phase "010" (higher p_0), the fill in order $+0, +1, -0$. More generally, valley U(1)×U(1) symmetry allows us to assign separately conserved fillings ν_+, ν_- to the two valleys. The two competing phases are defined by their fillings ν_+, ν_- (we write $\tilde{\nu} = \nu + 2$ in this regime):

$$(\nu_+, \nu_-)_{010} = \begin{cases} (2+\tilde{\nu}, 0) & \tilde{\nu} < 0 \\ (2, \tilde{\nu}) & \tilde{\nu} > 0 \end{cases} \tag{58}$$

$$(\nu_+, \nu_-)_{001} = \begin{cases} (1, 1+\tilde{\nu}) & \tilde{\nu} < 0 \\ (1+\tilde{\nu}, 1) & \tilde{\nu} > 0 \end{cases} \tag{59}$$

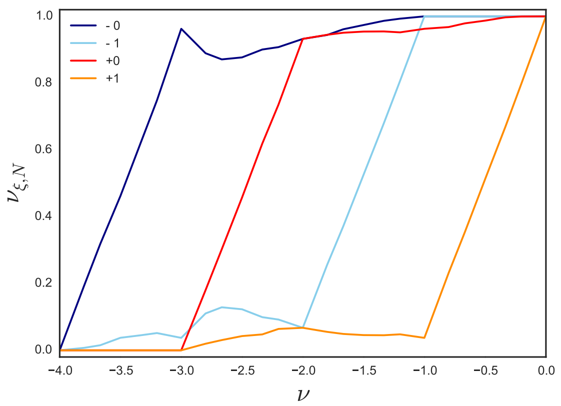

Supplementary Fig. 7. The densities $\nu_{\pm,N} = 2\pi\ell_B^2 \langle n_{\pm,N}\rangle$ (we assume spin-polarization for simplicity) as the total filling ν is increased. $p_0 < 0$ is small, so the orbitals fill in the order $-0, +0, -1, +1$. We see that while charge does fluctuate between $N = 0, 1$ orbitals of the same isospin, this deviation is at most about 10% of the filling. This explains why blue/red ($N = 0$) and cyan/orange ($N = 1$) appear as a sharp contrast in our C_A data.

Note that for fractional ν very close to the transition, there are presumably a multitude of phases in which *fractional* filling has transferred between the valleys, which we do not consider above. However, the current experiment isn't sensitive to these more delicate states, which are characterized by a lower energy scale, so they will appear as a small rounding of the phase boundary u_*.

The iDMRG conserves U(1)×U(1), so can be used to find the lowest energy state at filling (ν_+, ν_-) under the Hamiltonian $H = H^{(1)} + H^{(2)}$. While in principle the full Hamiltonian could be simulated in DMRG, for technical reasons it greatly simplifies matters to decompose the Hamiltonian as

$$H = \left[\sum_\xi \hat{N}_{\xi,1}\Delta_{10} + \mathbf{H}^{\mathbf{SU(4)}}\right]_\text{DMRG} + \left[\sum_{\xi,N} \frac{u}{2}\hat{N}_{\xi,N}\xi\alpha_N\right]_\text{P.T.} + \left[\mathbf{H}^\text{co} + \mathbf{H}^\mathbf{V}\right]_\text{H.F.} \tag{60}$$

The dominant part, $[\cdot]_\text{DMRG}$, will be evaluated in DMRG. The u-dependence $[\cdot]_\text{P.T.}$ is evaluated in first order perturbation theory, by evaluating $\langle \hat{N}_{\xi,N}\rangle$ using the ground states found in DMRG. When $\alpha_1 = 1$ first order perturbation theory is exact, since the layer polarization commutes with the Hamiltonian, and our tests indicate that more generally this introduces negligible error. We make this approximation so that $u_*(\nu)$ can be determined from a single DMRG run at $u = 0$, rather having to re-run DMRG for each value of u. Finally, $[\cdot]_\text{H.F.}$ is evaluated by neglecting inter-valley correlations, e.g. by taking $\langle n_+(r)n_-(r)\rangle \to \langle n_+(r)\rangle\langle n_-(r)\rangle$:

$$[\cdot]_\text{H.F.} = N_\Phi E_C \frac{d}{\ell_B}\left[\frac{1}{4}\frac{\epsilon_\text{BN}^\parallel}{\epsilon_\text{BLG}^\perp}(\nu_t - \nu_b)^2 + \nu_+\nu_-(-g_z + g_\perp\frac{1}{4})\right] \tag{61}$$

Since one of the two isospins is always at integer filling, and hence is largely inert, the inter-valley correlations are expected to be small (on top of the already small scale $\frac{d}{\ell_B}$). For example, at $\nu_+ = \nu_- = 1$, DMRG shows the pair correlation is around $\langle n_+(r)n_-(r)\rangle \sim 0.8\langle n_+(r)\rangle\langle n_-(r)\rangle$, a slight suppression from the Hartree value. Thus while largely negligible for determining the phase boundary u_*, effects of this form could be relevant for understanding the smaller energy scale governing spin physics, an interesting subject for future work.

All parameters *except* a_scr are those used or determined by the Hartree-Fock analysis. As will be discussed, we find that $a_\text{scr} = 0.22$ must be adjusted slightly to preserve the location of the $\nu = -2$ transition.

iDMRG numerics. In the infinite-DMRG method we place the quantum Hall problem $[\cdot]_\text{DMRG}$ on an infinitely long cylinder of circumference L, for which we compute the ground state energy density including the full effect of correlations. When both $2 > \nu_+, \nu_- > 0$ (for instance, in phase 001), it is necessary to keep *four* ZLL components in the iDMRG, $+0, +1, -0, -1$. This is because even when $\nu_\xi = 1$, isospin ξ acts as a polarizable medium due to the very small splitting between the $N = 0, 1$ orbitals. While computationally very expensive,[14] the ZLL orbital mixing is thus fully accounted for.

In principle H has a delicate ν-dependence sensitive to all the fractional competing phases (which may be distinguished at the level of 10^{-3} or $10^{-4}E_C$), which would require finite-scaling analysis to fully resolve. However, given the resolution of the

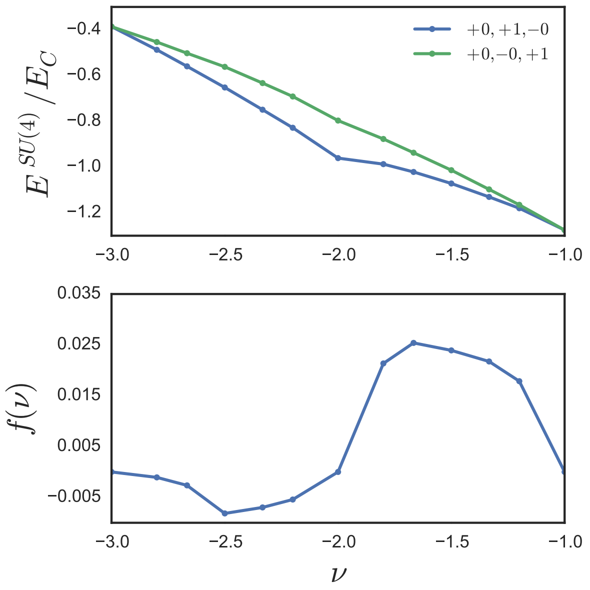

Supplementary Fig. 8. **a**) The SU(4)-symmetric part of the interaction energy for the two competing phases. **b**) The difference between these interaction energies, after subtracting out the linear contribution.

present experiment, we focus on the much larger and slowly varying background (at the level of $10^{-1}E_C$). For this purpose, we work on cylinders of circumference $L = 16\ell_B$ and use a DMRG-bond dimension of $\chi = 1600$, which results in an error in the energy per particle of around $10^{-4}E_C$, much smaller than the experimental features to be modeled. Specifying the valley fillings ν_+, ν_- according to Suppl. Eq. (59), iDMRG was used to compute the energy under $[\cdot]_{\text{DMRG}}$ for the two competing phases at $1+\tilde{\nu} \in \{0, 1/5, 1/3, 2/5, 1/2, 3/5, 2/3, 4/5, 1, \cdots, 2\}$.

Before discussing the resulting DMRG energies, we first address the issue of why blue/red and cyan/orange appear as distinct scales in the experimental C_{A} data, which requires that the $N = 0, 1$ levels to largely fill sequentially rather than as a mixture. Consider, for instance, the low $p_0 < 0$ phase in which (naively) the orbitals fill in the order $-0, +0, -1, +1$. With interactions, charge can fluctuate between the $N = 0, 1$ orbitals of the same valley. In Fig. 7, we show the iDMRG result for the densities $\nu_{\pm,N} = 2\pi\ell_B^2\langle n_{\pm,N}\rangle$ as the total filling $-4 < \nu < 0$ is varied. The deviation from the non-interacting expectation is at most around 10%, explaining the sharp contrast.

In Fig. 8a, we show the interacting part of the energy $E^{SU(4)}$ for both phases. The difference $\zeta = E_{010}^{SU(4)}(\nu = -2) - E_{001}^{SU(4)}(\nu = -2)$ is slightly smaller than its Hartree-Fock value, so to preserve the location of the transition we adjust the screening to $a_{\text{scr}} = 0.22$ (this adjusts Δ_{Lamb} accordingly). In Fig. 8b, we show their difference after extracting out the linear part, $f(\nu) = E_{010}^{SU(4)}(\nu) - E_{001}^{SU(4)}(\nu) - \zeta(1 - |\nu + 2|)$. The significantly different curvature on the two sides of $\nu = -2$ leads to the kink in the phase boundary discussed in the main text.

After calculating the DMRG and HF part of the energy at $u = 0$, the remaining polarization energy is determined from the DMRG expectation values $\frac{u}{2}\xi\alpha_N\langle n_{\xi N}\rangle$ shown in Fig. 7. From this we compute the boundary $u_*(\nu)$ shown in Fig. 3e-g.

SUPPLEMENTARY REFERENCES

[1] Jung, Jeil and MacDonald, Allan H. Accurate tight-binding models for the bands of bilayer graphene. *Phys. Rev. B* **89** (2014).

[2] Geick, R. and Perry, C. H. and Rupprecht, G. Normal Modes in Hexagonal Boron Nitride. *Physical Review* **146**, 543–547 (1966). URL http://link.aps.org/doi/10.1103/PhysRev.146.543.

[3] Ohba, Nobuko and Miwa, Kazutoshi and Nagasako, Naoyuki and Fukumoto, Atsuo. First-principles study on structural, dielectric, and dynamical properties for three BN polytypes. *Physical Review B* **63**, 115207 (2001). URL http://link.aps.org/doi/10.1103/PhysRevB.63.115207.

[4] Nandkishore, Rahul and Levitov, Leonid. Dynamical Screening and Excitonic Instability in Bilayer Graphene. *Phys. Rev. Lett.* **104** (2010).

[5] Gorbar, E. V. and Gusynin, V. P. and Miransky, V. A. and Shovkovy, I. A. Broken symmetry =0 quantum Hall states in bilayer graphene: Landau level mixing and dynamical screening. *Physical Review B* **85**, 235460 (2012). URL http://link.aps.org/doi/10.1103/PhysRevB.85.235460.

[6] Snizhko, Kyrylo and Cheianov, Vadim and Simon, Steven H. Importance of interband transitions for the fractional quantum Hall effect in bilayer graphene. *Physical Review B* **85**, 201415 (2012). URL http://link.aps.org/doi/10.1103/PhysRevB.85.201415.

[7] Papic, Z. and Abanin, D. A. Topological Phases in the Zeroth Landau Level of Bilayer Graphene. *Physical Review Letters* **112**, 046602 (2014). URL http://link.aps.org/doi/10.1103/PhysRevLett.112.046602.

[8] Shizuya, K. Structure and the Lamb-shift-like quantum splitting of the pseudo-zero-mode Landau levels in bilayer graphene. *Physical Review B* **86**, 045431 (2012). URL http://link.aps.org/doi/10.1103/PhysRevB.86.045431.

[9] Kharitonov, Maxim. Canted Antiferromagnetic Phase of the =0 Quantum Hall State in Bilayer Graphene. *Physical Review Letters* **109**, 046803 (2012). URL http://link.aps.org/doi/10.1103/PhysRevLett.109.046803.

[10] Sodemann, I. and MacDonald, A.H. Broken SU(4) Symmetry and the Fractional Quantum Hall Effect in Graphene. *Physical Review Letters* **112**, 126804 (2014). URL http://link.aps.org/doi/10.1103/PhysRevLett.112.126804.

[11] Lambert, J. and Cote, R. Quantum Hall ferromagnetic phases in the Landau level N=0 of a graphene bilayer. *Physical Review B* **87**, 115415 (2013). URL http://link.aps.org/doi/10.1103/PhysRevB.87.115415.

[12] Fano, G. and Ortolani, F. and Colombo, E. Configuration-interaction calculations on the fractional quantum Hall effect. *Physical Review B* **34**, 2670–2680 (1986). URL http://link.aps.org/doi/10.1103/PhysRevB.34.2670.

[13] Pezzini, S. and Cobaleda, C. and Piot, B. A. and Bellani, V. and Diez, E. Critical point for the canted antiferromagnetic to ferromagnetic phase transition at charge neutrality in bilayer graphene. *Physical Review B* **90**, 121404 (2014). URL http://link.aps.org/doi/10.1103/PhysRevB.90.121404.

[14] Zaletel, Michael P. and Mong, Roger S. K. and Pollmann, Frank and Rezayi, Edward H. Infinite density matrix renormalization group for multicomponent quantum Hall systems. *Physical Review B* **91** (2015). ArXiv: 1410.3861.
